# Combination of 5-fluorouracil and thymoquinone targets stem cell gene signature in colorectal cancer cells

**DOI:** 10.1038/s41419-019-1611-4

**Published:** 2019-05-16

**Authors:** Benardina Ndreshkjana, Aysun Çapci, Volker Klein, Pithi Chanvorachote, Julienne K. Muenzner, Kerstin Huebner, Sara Steinmann, Katharina Erlenbach-Wuensch, Carol I. Geppert, Abbas Agaimy, Farah Ballout, Chirine El-Baba, Hala Gali-Muhtasib, Adriana Vial Roehe, Arndt Hartmann, Svetlana B. Tsogoeva, Regine Schneider-Stock

**Affiliations:** 1 University Hospital, Friedrich-Alexander-University Erlangen-Nuremberg, Experimental Tumorpathology, Institute of Pathology, Erlangen, Germany; 2 University Hospital, Friedrich-Alexander University of Erlangen-Nuremberg, Institute of Pathology, Erlangen, Germany; 3 Organic Chemistry Chair I and Interdisciplinary Center for Molecular Materials (ICMM), Friedrich-Alexander University Erlangen-Nuremberg, Erlangen, Germany; 4 Department of Pharmacology and Physiology, Faculty of Pharmaceutical Sciences, Chulalongkorn University, Bangkok, Thailand; 5 Department of Biology and Center for Drug Discovery, American University of Beirut, Beirut, Lebanon; 6 Department of Pathology, Federal University of Health Sciences of Porto Alegre (UFCSPA), Porto Alegre, Brazil

**Keywords:** Cancer stem cells, Translational research

## Abstract

Cancer stem cells (CSCs) residing in colorectal cancer tissues have tumorigenic capacity and contribute to chemotherapeutic resistance and disease relapse. It is well known that the survival of colorectal CSCs after 5-fluorouracil (5-FU)-based therapy leads to cancer recurrence. Thus CSCs represent a promising drug target. Here, we designed and synthesized novel hybrid molecules linking 5-FU with the plant-derived compound thymoquinone (TQ) and tested the potential of individual compounds and their combination to eliminate colorectal CSCs. Both, Combi and SARB hybrid showed augmented cytotoxicity against colorectal cancer cells, but were non-toxic to organoids prepared from healthy murine small intestine. NanoString analysis revealed a unique signature of deregulated gene expression in response to the combination of TQ and 5-FU (Combi) and SARB treatment. Importantly, two principle stem cell regulatory pathways WNT/ß-Catenin and PI3K/AKT were found to be downregulated after Combi and hybrid treatment. Furthermore, both treatments strikingly eliminated CD133+ CSC population, accompanying the depleted self-renewal capacity by eradicating long-term propagated 3D tumor cell spheres at sub-toxic doses. In vivo xenografts on chicken eggs of SARB-treated HCT116 cells showed a prominent nuclear ß-Catenin and E-cadherin staining. This was in line with the reduced transcriptional activity of ß-Catenin and diminished cell adhesion under SARB exposure. In contrast to 5-FU, both, Combi and SARB treatment effectively reduced the angiogenic capacity of the remaining resistant tumor cells. Taken together, combination or hybridization of single compounds target simultaneously a broader spectrum of oncogenic pathways leading to an effective eradication of colorectal cancer cells.

## Introduction

Colorectal cancer (CRC) remains a leading cause of cancer-related deaths worldwide^1^. Two major obstacles in CRC therapy are the resistance of tumor cells to chemotherapeutic drugs and their relapse after treatment, limiting patient survival^2^. Accumulating evidence indicates that cancer stem cells (CSCs) are the driving force of cancer initiation, metastasis, recurrence, and they largely contribute to chemoresistance^3^. Recent studies showed that CSCs can be identified by the expression of CD133 cell surface marker. Indeed, CD133+ tumor cells are capable of seeding new tumors^4–7^. Also WNT/β-Catenin and PI3K/AKT signaling pathways play an essential role in stem cell maintenance and are associated with an enhanced tumorigenicity of CD133+ primary CRC cells^8^. The WNT cascade is the dominant force in controlling cell fate and the regulation of its key player ß-Catenin^9–11^. Blockage of WNT signaling was shown to inhibit angiogenesis and tumor growth in CRC^12^.

5-FU-based chemotherapy is the common choice for patients with CRC. Several combination strategies with plant-derived drugs have been proposed to overcome 5-FU resistance and to reduce its side effects^13–15^. We and others have demonstrated strong anticancer effects of thymoquinone (TQ), the main component of black seed^16–20^. Only a few combination studies with 5-FU have been reported supporting the chemosensitization effects of TQ^21,22^.

The generation of hybrids between natural products and conventional chemotherapeutics is a novel approach to obtain new anticancer compounds facilitating administration and allowing easier prediction of pharmacokinetic features^23–25^. In previous studies, we showed the importance of the hybridization concept by linking two natural compounds, TQ and artemisinin, along with other bioactive natural products being highly potent antimalarial, anticancer, and antiviral compounds^26–30^.

In this present study, we analyzed the effects of novel 5-FU/TQ hybrids in CRC cells in vitro and in vivo. A NanoString-based gene expression analysis was performed to compare the anticancer effect of the SARB hybrid with the effect of the individual compounds as well as Combi treatment. In comparison to the single drug treatments we identified additional novel targets exceeding the known mechanisms of action of 5-FU and TQ. We show that both combination strategies are highly effective against CD133+ CSC populations in CRC and simultaneously inhibit the WNT/ß-Catenin and PI3K/AKT signaling pathways. Thus, our findings strongly support the idea of combination therapy between the clinical drug 5-FU and the plant-derived compound TQ and suggest the hybridization concept as a promising strategy to develop new drug candidates for CRC.

## Results

### Generation of hybrid compounds of the natural product TQ (1) and 5-FU (2)

**Fig. 1:**
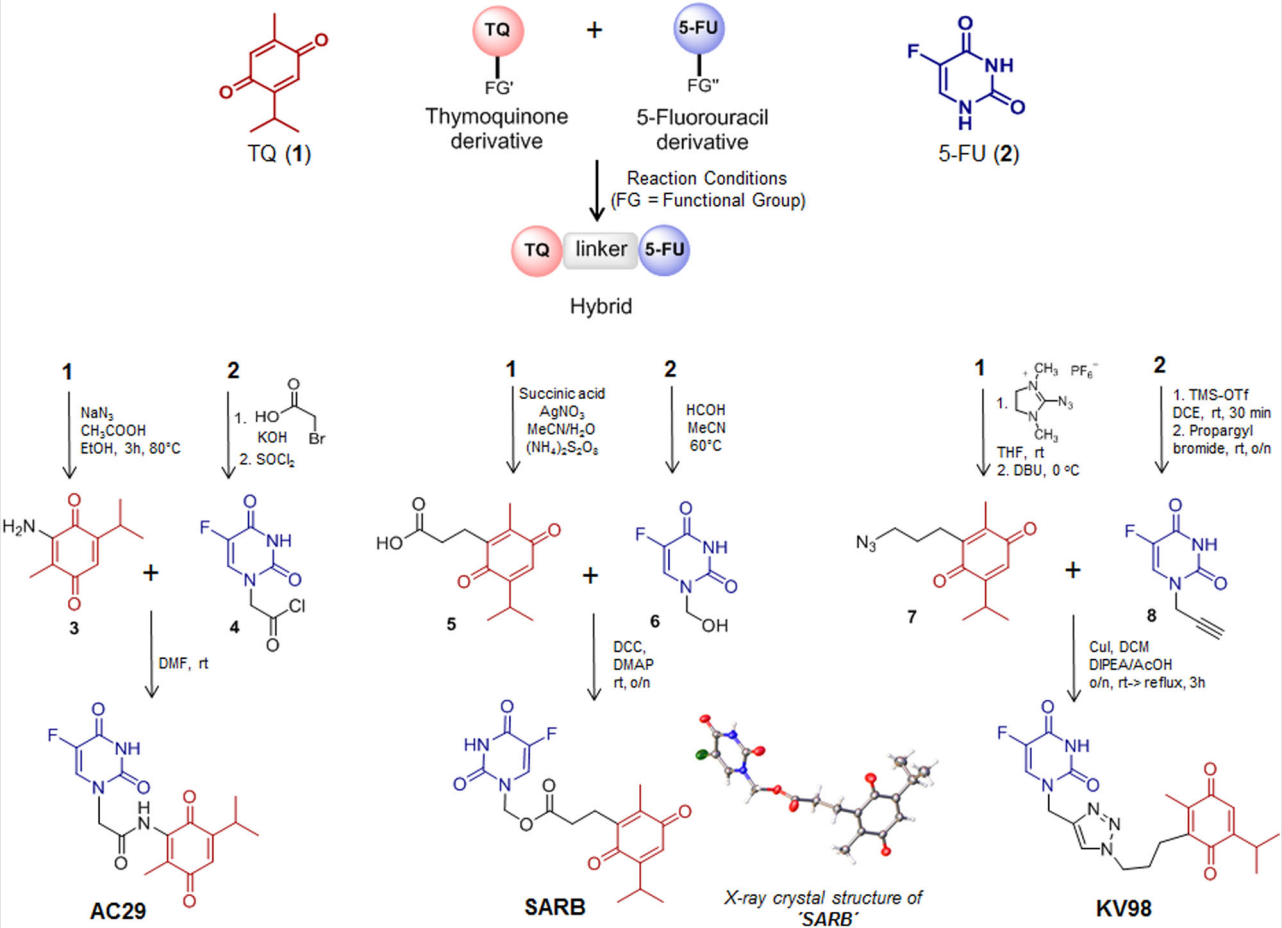
Generation of hybrid compounds from the natural product Thymoquinone (TQ, **1**) and 5-Fluorouracil (5-FU, **2**). All parent compounds (**3–8**) of hybrids (SARB, AC29, and KV98) were synthesized from Thymoquinone and 5-Fluorouracil

We designed hybrid molecules based on 5-FU and TQ. The parent compounds (**3–8**) of hybrids were synthesized from TQ and 5-FU. Hybrids were obtained via three different chemical reactions; esterification (SARB), amide coupling (AC29), and click reaction (KV98) (Fig. 1). All hybrid structures were identified with spectroscopic analysis and their purity was confirmed via elemental analysis (see supplementary information).

### Combination and hybrid treatments potentiate the cytotoxic effects of the individual compounds

First, we examined the anti-proliferative properties of the single drugs and their combination in a 1:1 mixture by exposing HCT116 and HT29 human colon cancer cells to different drug concentrations. We observed a clear reduction of viability in both cell lines, when treating with TQ or 5-FU alone, with HT29 cells being generally less sensitive (Suppl. Fig. 1A, B). The combination of TQ with 5-FU (Combi) increased the cytotoxic effect in HT29 cells (Suppl. Fig. 1C). Next, we synthesized three hybrids bearing variable linkers (Fig. 1). Interestingly, only SARB hybrid was effective in HCT116 cells with an IC_50_ = 16 µM, whereas the two other hybrids, KV98 and AC29, as well as SARB parent compounds, produced an IC_50_ > 100 µM (Suppl. Fig. 1D, E). Consequently, for all subsequent experiments we focused on SARB hybrid. When comparing Combi and SARB, both showed increased cytotoxicity in HCT116 with almost similar IC_50_, whereas in HT29 cells Combi produced a lower IC_50_ (Suppl. Fig. 1C, E). Next, we tested the cytotoxic effects of SARB against the HCEC nonmalignant colon epithelial cells. Intriguingly, SARB exerted higher IC_50_ values on normal cells (IC_50_ = 52 µM), suggesting specificity to cancer cells (Suppl. Fig. 1F). In order to evaluate the toxicity of the compounds under physiological condition, organoid cultures were treated with both individual compounds, Combi and SARB. In the control group, the organoids were found to gradually enlarge with a sharp morphology and pronounced villi. The organoid architecture was nearly unaffected after TQ treatment and the clear villi-lumen structure was only slightly disrupted under 5-FU, Combi, or SARB exposure at a dose up to 50 µM. Viability measurements showed only a minor reduction in viable cells to ~70–80% when individual treatments were used, while in response to the Combi and SARB, the vitality varied from 60% to 80% (Suppl. Fig. 2A, B). To determine whether the growth-inhibitory effect was related to the induction of apoptosis, we performed an Annexin/PI analysis. The cells were exposed to the IC_50_ of single compounds and IC_65_ of Combi and SARB. The apoptotic cell fraction was significantly increased when cells were treated with SARB in HCT116 cells; however, in HT29 cells the Combi and SARB exhibited comparable effects to the single drugs (Suppl. Fig. 2C, D). Altogether, these observations suggest the superior action of our designed drug combination strategy which we aimed to further investigate at the molecular level.

### Gene expression profiling identifies WNT/ß-Catenin and PI3K/AKT signaling pathways as the major targets of SARB hybrid treatment

To identify a specific pattern of gene alterations caused by single drugs, Combi or SARB, we performed a gene expression profiling using the NanoString PanCancer Panel. TQ-treated and 5-FU-treated cells displayed a different gene expression profile compared to the untreated control as shown in the scatter plot diagram, where each point represents a particular gene (Suppl. Fig. 3A). Using STRING database we found that the affected downregulated genes under TQ belong to the RAS, RAP1, PI3K, and WNT signaling pathways (Suppl. Fig. 3B), as previously described^31–33^. The most significantly altered pathways in TQ upregulated genes are given in Suppl. Fig. 3C and include mostly the MAPK, PI3K, focal adhesion, and p53 signaling pathways. As expected, 5-FU downregulated components of the cell cycle pathway (Suppl. Fig. 3D) and led to the upregulation of p53-target genes associated with DNA damage response (GADD45A), cell cycle regulation (CDKN1A), and intrinsic apoptotic signaling (FAS, BAX, PML, SFN, EPHA2). The most significantly altered upregulated pathways in 5-FU are given in Suppl. Fig. 3E.

**Fig. 2:**
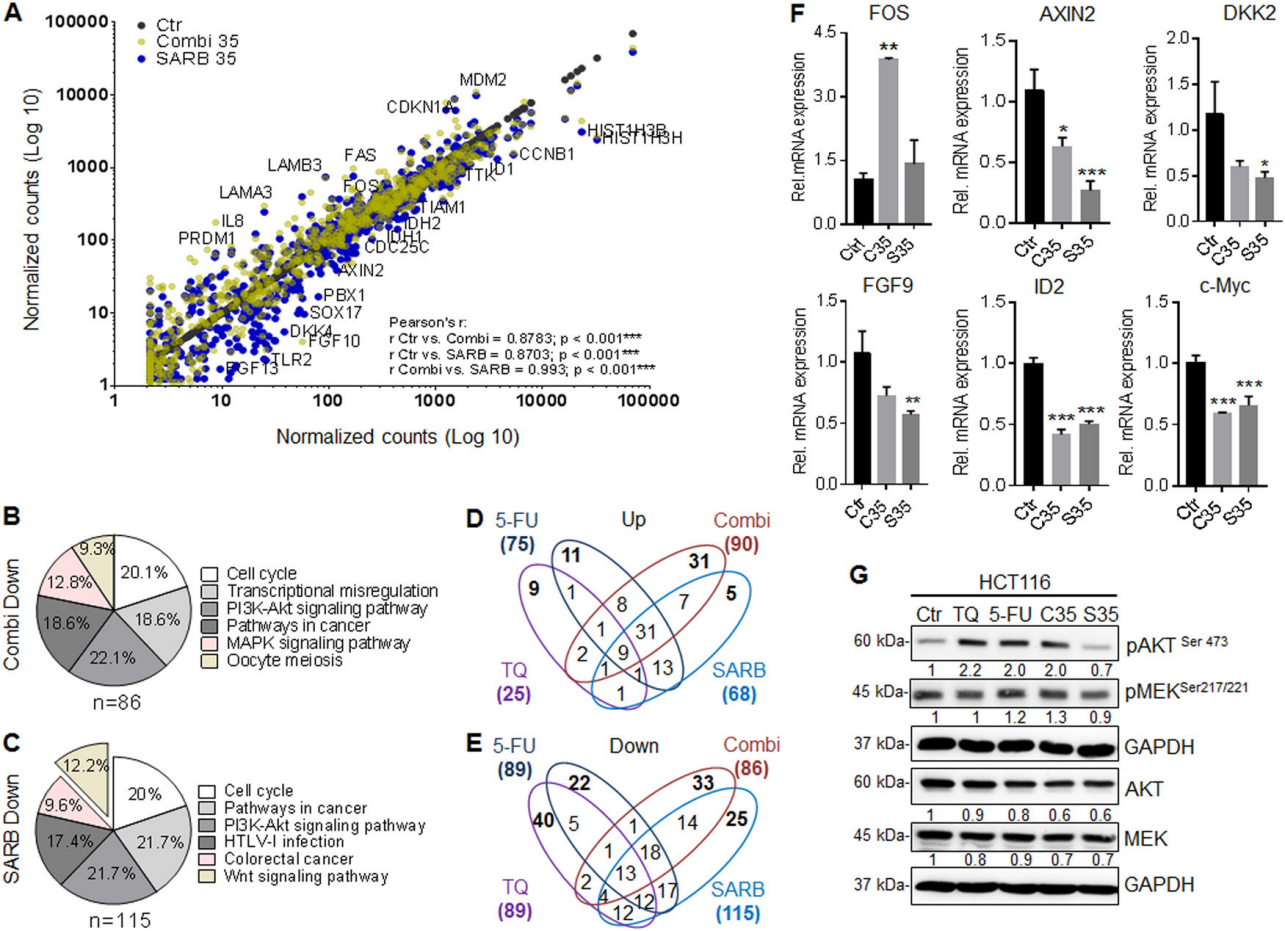
Gene expression profiling identifies WNT/ß-Catenin and PI3K/AKT signaling pathways as the major targets of SARB hybrid treatment. (**A**) Scatter plot of the whole NanoString gene set of HCT116 cells after 24 h of treatment with Combi and SARB hybrid. Normalized grouped data are shown. Log_10_ data are shown as treatment vs. untreated samples (*n* = 3). Pearson's correlation was calculated using GraphPad Prism 7.0. KEGG pathway was performed using STRING database (version 10.5) on the significantly downregulated genes under (**B**) Combi and under (**C**) SARB treatment. (**D**) Venn diagram of significantly up and (**E**) downregulated genes upon treatments. A *p*-value of  ≤ 0.05 and a fold change of  ≥ / ≤ 1.5-fold are selected for the analysis. (**F**) RT-qPCR validation of WNT target genes and FOS gene in HCT116 cells. Gene expression fold-change differences in Combi and SARB hybrid after 24 h of treatment. Values represent means ± SEM (*n* = 3), (*p < 0.05; **p < 0.01; ***p < 0.001; One way-ANOVA). B2M was used as a housekeeping gene. (**G**) Western Blot analysis of key proteins involved in PI3K/AKT signaling in HCT116 cells after 24 h of treatment with TQ 40, 5-FU 15, Combi, and SARB at same concentration of 35 µM. Western Blot images are representative of at least two independent experiments. The quantification values are presented as relative to GAPDH housekeeping protein

Compared to the individual treatments, the Combi treatment reinforced the downregulation of NGFR, NPM1, HIST13H, JUN, and PLA2G4C in a possible additive manner (Suppl. Table 1). Considering the upregulated genes in Combi, we found a remarkable higher frequency of potentially synergistic effects detectable for SPP1, EGF, SPRY1, and IGFBP3. In case of the FGF signaling antagonist SPRY1 and the metastasis suppressor gene IGFBP3^34^, it seems to be an inhibition of an oncogenic pathway whereas the upregulation of the two secreted factors SPP1 and EGF seems to be an undesired side effect of treatment reinforcing the PI3K signaling. Nevertheless, we identified a specific gene target panel whose expression was altered by Combi treatment including genes, such as FST, PLA2G10, NGF, PAK7 DNMT1, and BIRC3. Although many genes were deregulated in same direction as observed for the single treatments, we provide evidence that the combination treatment also targets a characteristic gene profile (Suppl. Table 1). Next, in a scatter plot, we confirmed dissimilarities among SARB and Combi-treated samples, especially in the downregulated genes (Fig. 2A).

Although KEGG pathway analysis of the downregulated genes revealed a significant cell cycle inhibition for both treatments, there was a specific set of downregulated WNT-associated genes only in the SARB-treatment group (Fig. 2B, C). We believe, this signature derives from TQ, since we could confirm the respective panel of genes with WN16, TBL1XR1, RAC1, PPP3CA, PPP3CB, PPKACB, and CTNNB1 both significantly downregulated under TQ and SARB treatment. The upregulated genes mostly affected the PI3K/AKT, focal adhesion, and p53 pathways (Suppl. Fig. 3C, E, G).

To identify specific gene signatures, we set another strong selection criterion by overlapping all the treatments and focusing on the uniquely deregulated genes. From 89 downregulated genes in TQ and 5-FU, 31 genes were overlapping, whereas in 25 and 75 upregulated genes in TQ and 5-FU, respectively, 12 genes were similarly altered (Fig. 2D, E). We could reduce the target panel to 25 downregulated and 5 upregulated genes that were uniquely and significantly altered in SARB-treated samples (Fig. 2D, E). When examining these 25 genes by KEGG pathway analysis, we distinguished another key oncogenic pathway, the PI3K pathway, as the most downregulated pathway under SARB exposure. These unique 30 genes in SARB are given in Suppl. Table 2. Surprisingly, we identified the stress oncogene FOS to be massively upregulated under Combi and SARB did not alter its expression, which was confirmed by RT-qPCR (Fig. 2F).

Next, we validated the unique WNT and PI3K/AKT gene signature obtained by NanoString analysis using RT-qPCR and Western Blot analysis. We verified a reduced expression of AXIN2, the most prominent transcriptional target of the canonical WNT signaling, as well as other WNT target genes such as DKK2, FGF9, ID2, and c-Myc (Fig. 2F). Interestingly, Combi treatment also affected the WNT pathway; however, with SARB being more effective in suppressing the three oncogenes AXIN2, DKK2, FGF9. Regarding the PI3K/AKT pathway we selected 16 genes that were significantly downregulated after SARB treatment whereas only four of them were deregulated in the same manner after Combi treatment (Suppl. Table 3).

To validate that the changes in the gene expression pattern affect also the protein level, we investigated treatment effects on the key players of the PI3K/AKT pathway. All treatments did not show strong effects on the total AKT and MEK levels. Individual compounds and Combi led to increased levels of pAKT and pMEK, whereas SARB did not target or even slightly downregulated these proteins suggesting that the hybridization strategy is superior to the single or Combi treatments at this point (Fig. 2G).

**Fig. 3:**
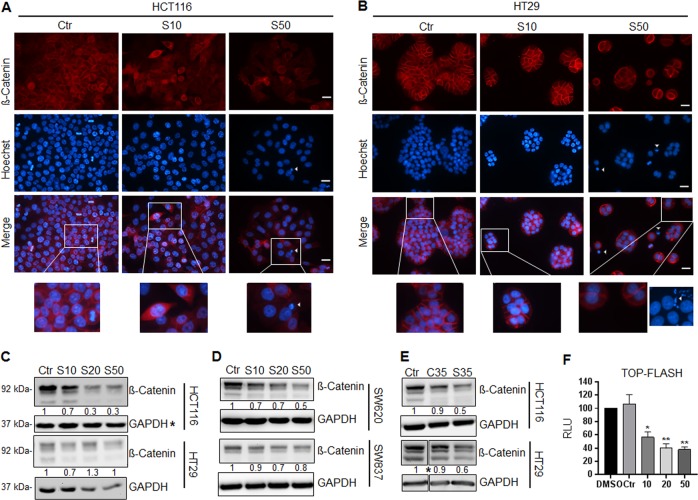
WNT/ß-Catenin signaling pathway is a major target of SARB hybrid treatment. (**A**) Representative images of immunofluorescence staining of ß-Catenin in HCT116 cells after treatment for 48 h with SARB 10 and 50 µM. Scale bar—25 µm. The lower panel shows images for the control, SARB 10 µM, and 50 µM group which were digitally enlarged. (**B**) Representative images of immunofluorescence staining of ß-Catenin in HT29 cells after treatment for 48 h with SARB 10 and 50 µM. Scale bar—25 µm. The lower panel shows images for the control, SARB 10 and 50 µM group which were digitally enlarged. An additional image (derived from another replicate) of Hoechst-stained cells was added to this panel to show the presence of micronuclei when treated with 50 µM of SARB. All images are representative for at least two independent experiments. (**C**) Western Blot analysis of ß-Catenin in HCT116 and HT29 cells treated with different concentrations of SARB for 48 h. The star (*) indicates that the GAPDH blot is the same as in Fig. 5f. (**D**) Western Blot analysis of ß-Catenin in SW620 and SW837 cells treated with different concentrations of SARB for 48 h. (**E**) Western Blot analysis of ß-Catenin in HCT116 and HT29 cells treated with 35 µM of Combi or SARB for 48 h. The star (*) indicates that two other treatments were excluded from this blot. The entire original blot is given in supplementary information. Western Blot images are representative for at least two independent experiments. GAPDH immunoblots served as loading control. (**F**) HCT116 cells transfected with TOP-FOP-FLASH and ß-galactosidase either treated with SARB for 24 h or left untreated and the data is shown as a percentage relative to DMSO. The reporter assay results represent the average of two independent transfection experiments. RLU relative light units

Since nuclear localization of ß-Catenin reflects an activated WNT pathway, we investigated the ß-Catenin distribution in HCT116 and HT29 cells before and after treatment with SARB. HCT116 and HT29 untreated cells showed nuclear ß-Catenin localization in a sub-population of cells in addition to pronounced membranous and cytosolic localization. Interestingly, when treating HCT116 cells with low concentrations of SARB (10 µM), ß-Catenin was expressed in apoptotic nuclei with condensed chromatin and cells gain a more spindle cell-like phenotype, an effect that was lost upon treatment with 50 µM SARB (Fig. 3A). SARB treatment had no significant effect on cell migration, which supports our findings of no prominent alteration of cell differentiation (Suppl. Fig. 4A). ß-Catenin was mostly lost from the plasma membrane of the surviving population of HCT116 cells treated with SARB, suggesting that SARB might selectively disrupt the cell–cell contact in these cells. This effect was less pronounced in HT29 cells where cell aggregates were still visible possibly leading to the higher resistance against SARB (Fig. 3B). When evaluating the NanoString data for the available 30 ECM and adhesion-associated genes we extracted six significantlly upregulated genes (>1.5 fold; *p*-value ≤ 0.05), i.e. ITGA6, NOTCH1, LAMA3, LAMB3, THBS1, TGFB1 for SARB treatment.

Interestingly, both cell lines showed nuclear anomalities visible as micronuclei or nuclear buds, a phenomenon described by Nersesyan et al. ^35^ as a genotoxic effect (Fig. 3A, B). SARB effectively decreased the ß-Catenin levels in different colorectal tumor cell lines HCT116, HT29, SW620, and SW837 (Fig. 3C, D) and seemed to be more effective than Combi (Fig. 3E). This decrease in ß-Catenin protein levels was accompanied by a significant reduction in transcriptional activity of ß-Catenin using a TCF/LEF reporter plasmid (Fig. 3F), suggesting that SARB hybrid has the capability to interfere with WNT/ß-Catenin signaling and to block its transcriptional activity.

### CD133 is targeted by Combi and SARB hybrid treatments

**Fig. 4:**
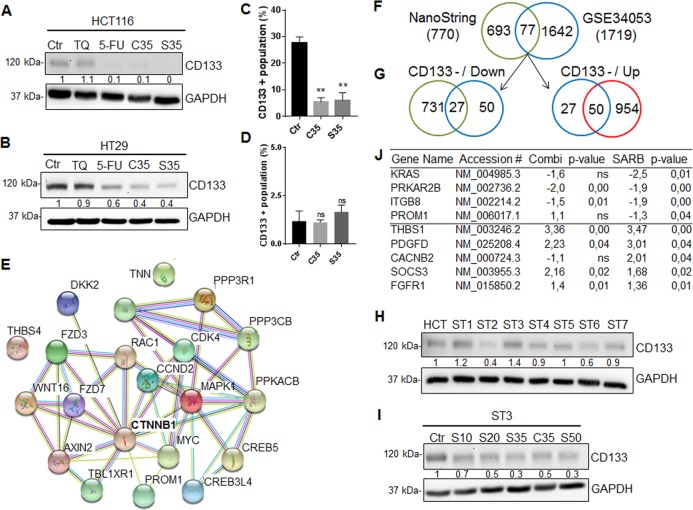
CD133 is targeted by the Combi and SARB hybrid treatments. (**A**) Western Blot analysis of CD133 marker in HCT116 and (**B**) in HT29 cells after 48 h of treatment with TQ 40, 5-FU 15, Combi, and SARB 35 µM. (**C**) FACS analysis of CD133 population after treatment of HCT116 cells and (**D**) HT29 cells with Combi and SARB for 48 h. Error bars indicate SEM calculated from three and two independent experiments for HCT116 and HT29, respectively. (**E**) Protein–protein interaction of the genes which were downregulated under SARB and which belong to PI3K/AKT and WNT signaling pathways generated via STRING. (**F**) Venn diagram of GEO dataset overlapping with the NanoString PanCancer Pathway Panel dataset. All 770 genes were used from NanoString. (**G**) Venn diagram of the 77 overlapping genes from (**f**) with the downregulated genes (green line) and upregulated genes (red line) in CD133− population deriving from the GEO dataset. (**J**) Table with the four downregulated overlapping genes and five upregulated overlapping genes in Combi and SARB treatment.(**H**) Western Blot analysis of CD133 in HCT116 parental cells and the seven HCT116 CSC enriched-sub-clones. (**I**) Western Blot analysis of CD133 expression in ST3 clone after 48 h of treatment with Combi and SARB at different drug concentrations. GAPDH immunoblots served as a loading control. All Western Blot images are representative for at least two independent experiments

Since SARB targeted the CSC-related WNT/ß-Catenin and PI3K/AKT-signaling pathways^36^, we next focused on the most common stem cell marker in CRC, namely CD133 (PROM1), which was found to be significantly down-regulated by 5-FU, Combi, and SARB treatment in both cell lines (Fig. 4A, B). FACS analysis confirmed the significant reduction in CD133-positive tumor cell population after Combi and SARB treatment of HCT116 cells (Fig. 4C). Using our FACS-specific CD133 antibody, HT29 cells expressed CD133 only in <2% of cells (Fig. 4D). In SW620 cells we did not see any dose-dependent effect of SARB on CD133 expression and the rectal SW837 cells did not express CD133 at all (data not shown). Next, when PROM1 was additionally included in the STRING PI3K/AKT/WNT network, it showed a direct interaction with ß-Catenin (CTNNB1) (Fig. 4E).

We next used the public GEO database (GSE34053^37^) to extract specific gene expression profiles of CD133+ and CD133− colorectal tumor cells. When matching the gene panel of GSE34053 with our NanoString panel, we could acquire 77 genes that were included in both setups (Fig. 4F). We hypothesized that the gene signature after SARB treatment might resemble the gene signature of the CD133− population. From the 77 common genes, 27 genes were overlapping with the downregulated genes in the CD133− population (Fig. 4G). Four of these 27 genes were significantly downregulated also in SARB and included the oncogene KRAS, the membranous protein kinase PRKAR2B, which is known to regulate the MAP2 kinase, the receptor for fibronectin ITGB8 and PROM1 (Fig. 4J). From this panel, only two genes were significantly downregulated in Combi. A similar result was achieved when comparing upregulated genes in CD133− and HCT116 cells under SARB exposure with four genes, including inhibitor of angiogenesis THBS1 and PDGFD, which plays an important role in wound healing by macrophage recruitment, calcium channel protein CACNB2, and SOCS3, an inhibitor of the JAK/STAT pathway (Fig. 4j). Thus, we hypothesize that the two combination modalities, Combi and SARB treatments, might provide a selective advantage against CD133-enriched tumor populations. To study this, we analyzed CD133-enriched HCT116 clones derived from spheroids by single cell dilution strategy as previously described^38^ (Fig. 4H). Indeed, Combi and SARB treatments were highly effective in eradicating the CD133+ population in the CD133-enriched clone 3 (Fig. 4I).

### SARB hybrid targets self-renewal capacity of CRC cells

Since WNT and PI3K/AKT-abrogation is known to decrease the stem-cell like features of tumor cells we evaluated the effect of SARB in sub-toxic doses using the sphere propagation assay (Supp. Fig. 5A). Here, HCT116 cells were continuously exposed to different sub-doses of SARB hybrid immediately after plating the cells. Impressively, we found that even a low dose of 3 µM SARB was enough to significantly reduce the propagated spheres already at the first generation, and spheroids were even completely eradicated at 6 and 10 µM (Suppl. Fig. 5B, C). When following sphere formation over time, a dose as low as 1 µM SARB was capable to decrease sphere numbers by more than two-fold, however not significantly (Suppl. Fig. 5D–G).

**Fig. 5:**
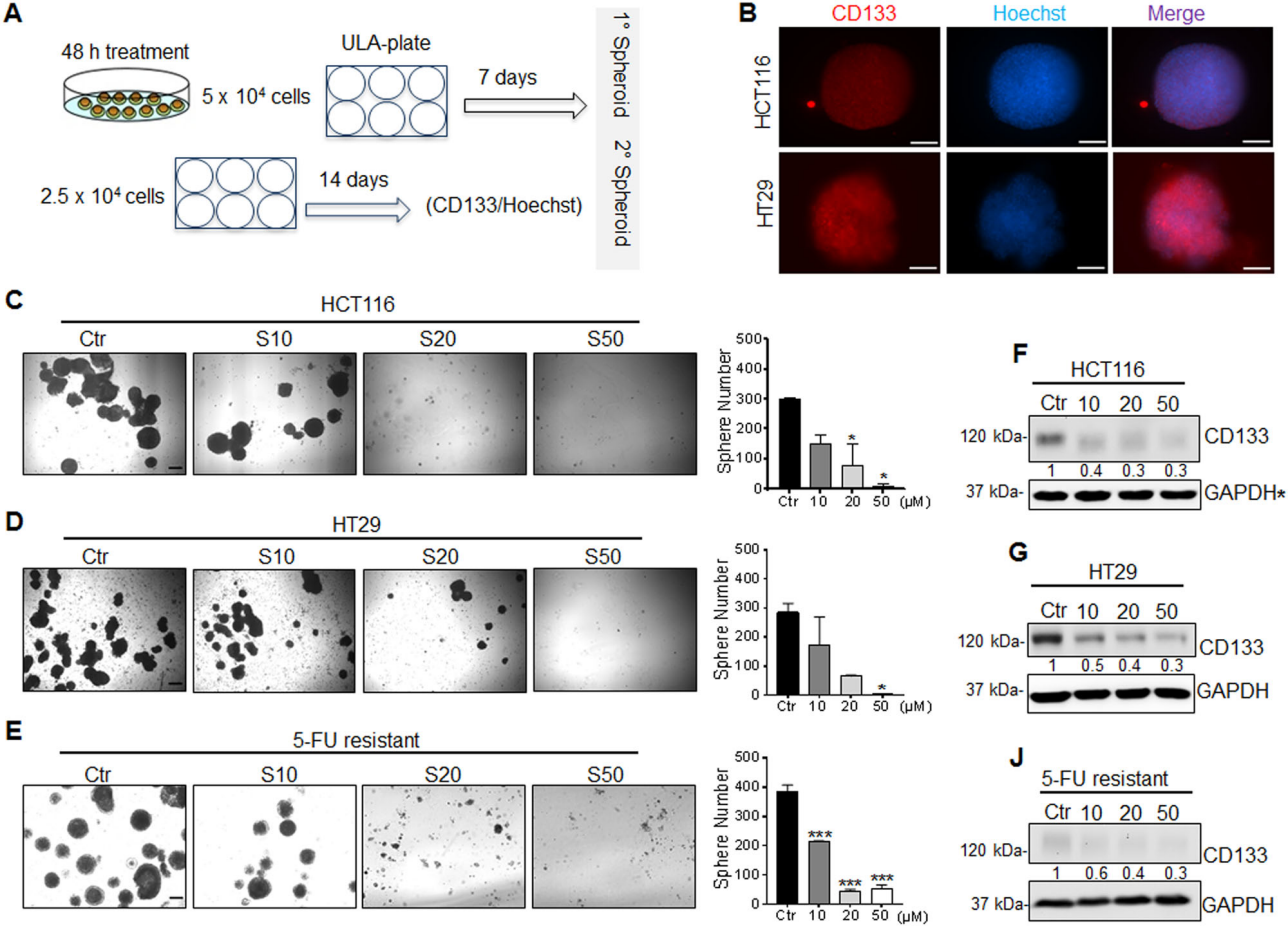
SARB hybrid targets self-renewal capacity of CRC cells. (**A**) Schematic overview of the protocol used for spheroid formation assay (SFA). (**B**) Representative immunofluorescence images of CD133 staining (red) in 3D HCT116 and HT29 cells (*n* ≥ 2). Hoechst staining (blue) is used to stain the nuclei. Scale bar—100 µm. Representative bright-field images of spheroids in the control group and in the treated ones with increasing concentration of SARB in HCT116 (**C**), HT29 (**D**) and 5-FU resistant cells (**E**). Scale bar—250 µm. Sphere number quantification in the control and SARB-treated groups. Data represent the mean ± SEM from two independent experiments (*p < 0.05; **p < 0.01; ***p < 0.001; One way-ANOVA). Western blot analysis of CD133 in (**F**) HCT116, (**G**) HT29, and (**J**) 5-FU-resistant cells after SARB treatment. GAPDH served as a loading control. Western Blot images are representative for at least two independent experiments

In a second approach, we studied the ability of HCT116, HT29, and 5-FU-resistant HCT116 cells to form 3D non-adherent spheroids under different drug pre-treatments (Fig. 5A). As expected for this model, HCT116 and HT29-derived 3D spheres showed enrichment in the CD133 CSC marker when compared to HCT116 cells grown in 2D (Fig. 5B). When CRC cells were pretreated with different concentrations of SARB, we observed a clear dose-dependent attenuation of CSC-derived spheroids for all three cell lines (Fig. 5C–E). As expected, 5-FU-resistant cells showed the highest ability to form spheres (Fig. 5E). 5-FU treatment of HCT116 cells led to the formation of a low number of larger spheres, whereas Combi treatment at 35 µM was as effective as SARB treatment at the same concentration (Suppl. Fig. 6A). SARB treatment led to a dose-dependent decrease in CD133 protein levels in all three CRC lines (Fig. 5F–J).

### SARB hybrid diminishes the in vivo growth of CAM xenografts

**Fig. 6:**
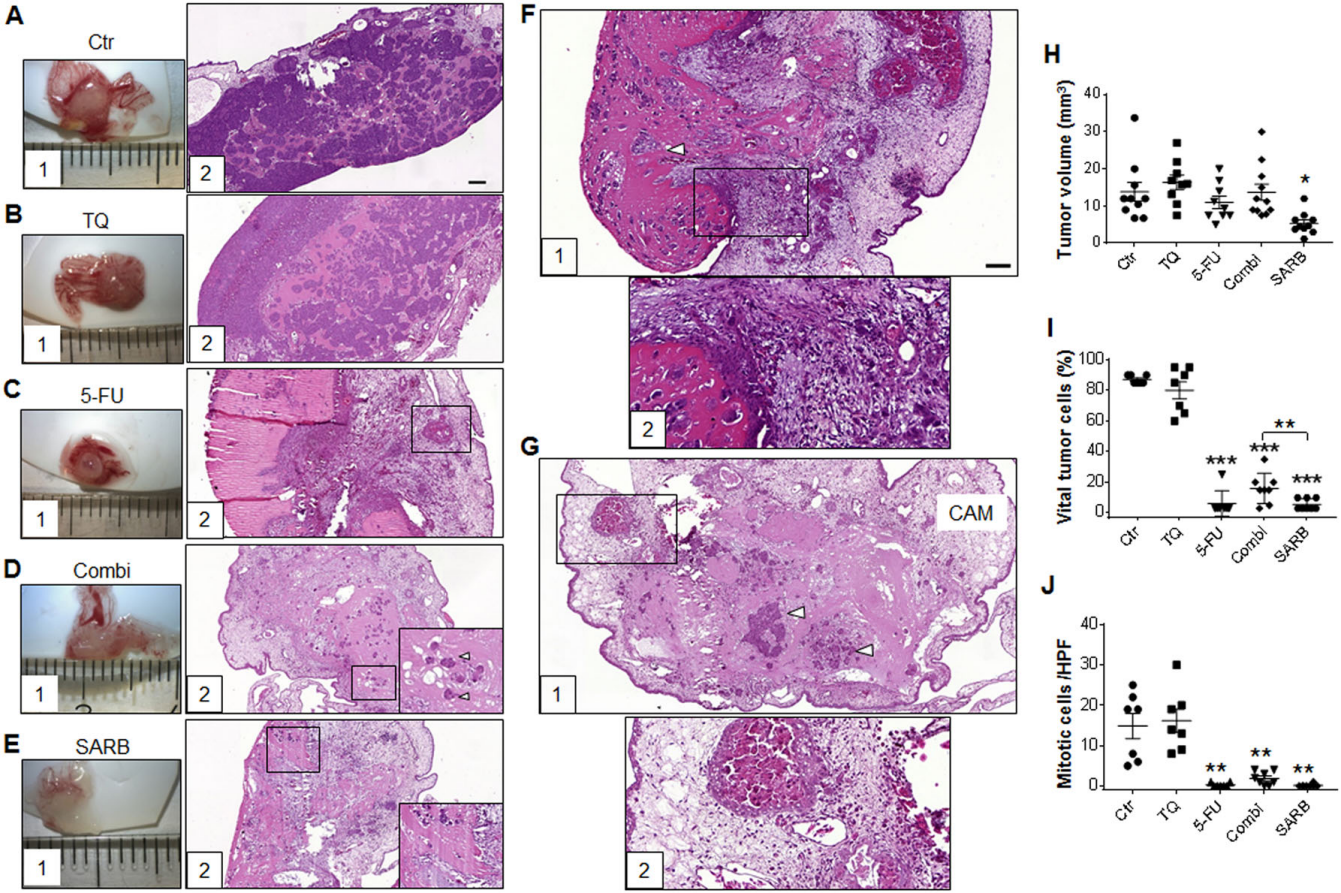
SARB hybrid diminishes the in vivo growth of CAM xenografts. (**A1**–**E1**) Representative light microscopy images of CAM tumors derived from untreated (control) and pre-treated (single drugs, Combi and SARB) HCT116 cells and the respective H&E histological staining (**A2**–**E2**, ×10). The ruler segments represent a length of 1 mm. (**D2**, **E2**) Computer-enlarged images of H&E staining’s show a rest of mitotic cells after combination treatment (white arrows). H&E staining of micrografts derived from Combi pre-treated cells (**F1**, ×10) and (**F2**, ×40). H&E staining of micrografts derived from SARB pre-treated cells (**G1**, ×10) and (**G2**, ×40). White arrows indicate areas of vital tumor cells, rosa color represents Matrigel. (**H**) Tumor volume calculation in HCT116 CAM samples (*p < 0.05; One way-ANOVA). (**I**) Fraction of vital tumor cells and (**J**) evaluation of mitotic activity in CAM sections. Mitosis in the vital tumor areas (average of five HPFs) (*p < 0.05; **p < 0.01; ***p < 0.001; One way-ANOVA) is given. Mann–Whitney *t*-test was used for comparison of Combi and SARB (*n* ≥ 7). Scale bar—100 µm

In a next step, we studied the effects of all compounds in vivo by employing the CAM assay, a model that has been widely established for drug screening^39^. Tumor cells were treated prior to CAM transfer since we hypothesize that targeting CSCs should reduce the potential to develop vital tumors. Ex ovo and histological images of HCT116 xenografts pretreated with the drugs for 48 h are presented in Fig. 6A1–E2. We observed that HCT116 control cells developed substantially well-vascularized tumor masses, while tumor xenografts from 5-FU, Combi, and SARB pre-treated cells showed extended areas of dead cells and calcification. TQ-treated cells formed rather well growing vital tumors. Comparing vessel infiltration into the tumor cell matrix, we observed a high vessel density with intense bleeding in 5-FU group (Fig. 6C2), whereas vascularization in CAM tumors of Combi or SARB pre-treated HCT116 cells was relatively low or moderate (Figs. 6F1, F2, G1, G2). The size of CAM tumors derived from SARB pre-treated cells was strikingly smaller when compared to the control group, whereas all other treatments did not show any significant effects on the tumor volume (Fig. 6H). There were significantly reduced numbers of vital tumor cells in 5-FU, Combi, and SARB-treatment groups, where SARB seemed to be more effective than Combi (Fig. 6I). In contrast to SARB, Combi pre-treated tumors showed an increased peritumoral lymphoplasmocytic infiltration, some immune cells and mild fibrosis (Figs. 6F1, F2).

**Fig. 7:**
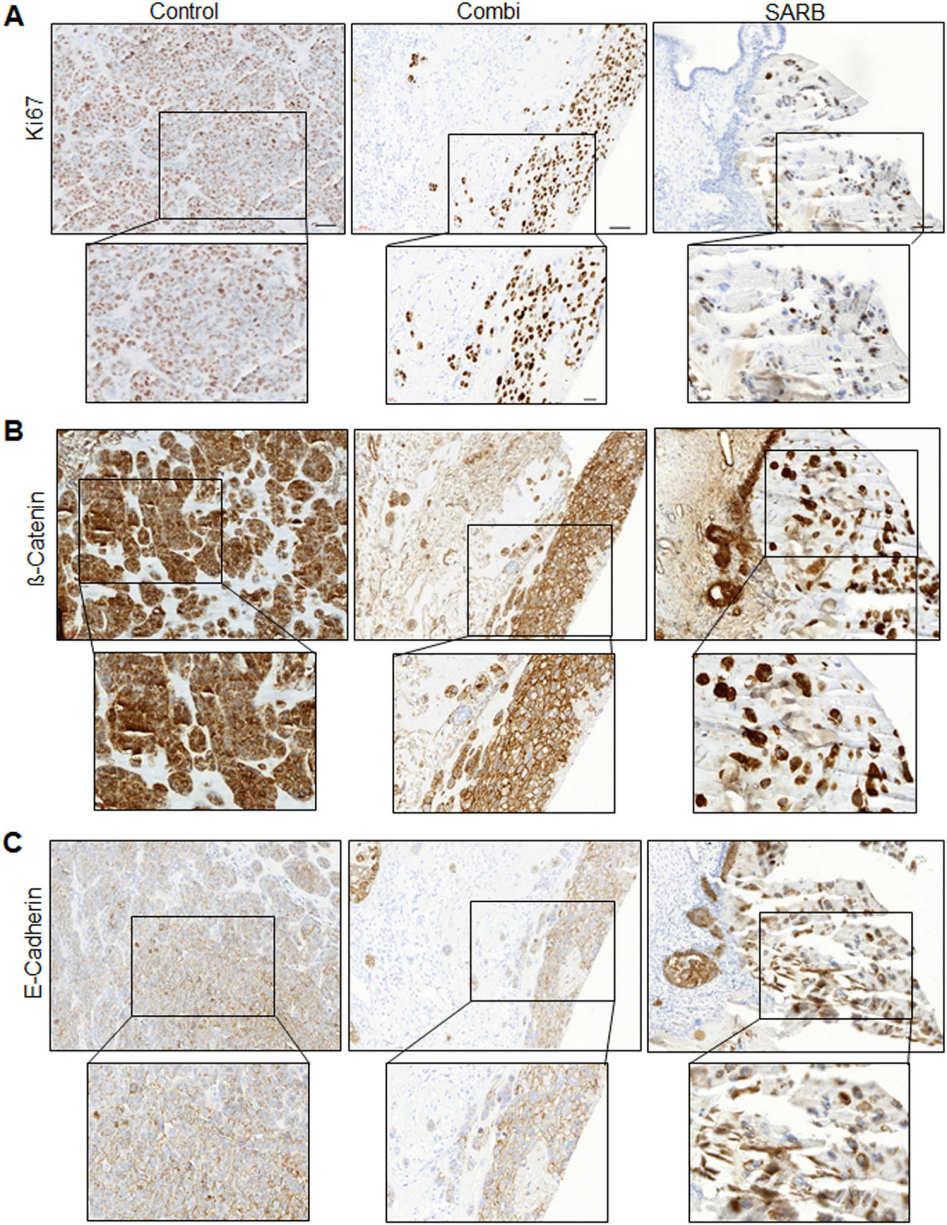
Combination treatments affect proliferation and adhesion in vivo. Immunohistochemical stainings of (**A**) Ki67, (**B**) ß-Catenin, and (**C**) E-cadherin of CAM-xenografts derived from control, Combi and SARB-treated HCT116 tumor cells. Upper panel shows ×20 magnifications; Scale bar—50 µm. Lower panel shows ×40 magnifications; Scale bar—20 µm

When quantifying the number of mitoses, we observed cells that were still proliferating in the Combi-derived xenografts, whereas SARB-derived tumors basically did not show any sign of mitotic activity (Fig. 6D2, E2, J), but Ki67 staining verified vital proliferating cells in both, Combi and SARB-treated xenografts (Fig. 7A). When qualitatively evaluating ß-Catenin staining pattern we observed a more membranous distribution after Combi and a more prominent nuclear ß-Catenin staining after SARB treatment (Fig. 7B). To assess a possible drug-dependent EMT induction we stained CAM xenografts with E-cadherin. In general the E-cadherin levels did not remakably change after Combi and SARB treatment (Fig. 7C), an effect that we also confirmed in Western Blotting of 2D cell culture (data not shown). Interestingly, E-cadherin staining after Combi treatment was mainly visible in the membrane and cytoplasm, whereas SARB treatment resulted in a mostly nuclear pattern, especially in single cells and not when cells were clustered (Fig. 7C).

## Discussion

Acquired therapeutic resistance of tumor cells contributes to severe clinical restrictions in CRC. An additional limitation is the fact that many available drugs target the fast dividing cells and not the CSCs, the drivers of post chemotherapy recurrence^40^. Recent reports showed that 5-FU treatment might even selectively enrich a subset of CSCs^41,42^. Here natural plant-derived compounds, such as TQ, could be potentially promising^43^. Indeed, TQ treatment potentiates the effects of conventional drugs in various cancers^17,44^.

We extended our study from single and Combi treatments to the treatment with new hybrid compounds. We identified the SARB hybrid as the most promising compound against CSC-like cells. Although SARB showed similar antitumor effects as the Combi treatment in vitro, it was more effective in vivo in the CAM assay. Combi and SARB showed commonly deregulated genes of the cell cycle and PI3K pathway, an oncogenic trigger that is closely associated with tumor aggressiveness^45^. One drawback for Combi treatment was the massive up-regulation of the proto-oncogene FOS, which in contrast was not affected under SARB exposure. Moreover, we uncovered the WNT signaling pathway as the distinctive pathway targeted only by SARB. Although this WNT effect appears to arise from TQ mediated signaling, it is not targeted by Combi treatment in the same manner. Target genes AXIN2, DKK2 and FGF9, well known for their potential to promote tumor formation and progression in vitro as well as in vivo in CRC, were significantly downregulated in response to SARB^46,47^.

ß-Catenin has a bidirectional role also as cell adhesion molecule at the plasma membrane via interaction with E-Cadherin. Thus, SARB-induced loss of ß-Catenin together with loss of E-cadherin at the plasma membrane might be associated with loss of cell–cell contact thus diminishing cell adhesion. We selected six deregulated genes that are associated with ECM and cell adhesion. The significance of this gene panel is discussed very controversy in CRC. As an example, THBSP1 decreased tumor cell adhesion via promotion of the urokinase plasminogen activator^48^. In contrast, THBSP1 can also override these intrinsic pro-adhesive effects, promoting the detachment of tumor cells from primary tumor. Since these six genes were deregulated in both Combi and SARB treatment group, their function is context/stimulus dependent or might be not responsible for the observed effect on cell–cell adhesion at all. Since migration capability and E-cadherin levels were unaffected by SARB, we exclude dramatic alterations in cell plasticity and strongly believe that the tumor simply dissociates. This is also in agreement with the inability of tumor cells to form spheroids or xenografts after SARB treatment. The tumor grafts appear as single distributed cells with prominent nuclear E-cadherin and ß-Catenin. In a first view ß-Catenin shuttling to the nucleus would insist on an activation of WNT signaling. However, nuclear E-cadherin is a potent inhibitor of ß-Catenin transcriptional activity^49^, which we confirmed by a reduction of ß-Catenin on its TCF-responsive genes after SARB treatment. The higher resistance to SARB in HT29 cells or in the surviving cell clusters in HCT116 xenografts of Combi treatment could be caused at least partially by the consistent level of ß-Catenin in the cell membranes. Although the upregulation of the cell cycle inhibitor p21 after Combi and SARB treatment might suggest an induction of senescence-like phenotype, we found that in CAM tumors the survived cells were Ki67 positive.

Another stem cell-related pathway, the PI3K/AKT pathway, was uncovered to be downregulated under SARB^50^. The key players of this pathway, such as pAKT, AKT, and MEK proteins were found to be slightly downregulated under SARB treatment. More convincingly 16 genes associated with the PI3K/AKT pathway extracted from the NanoString panel were deregulated under SARB exposure but only four of them under Combi. Conversely, the single compounds and Combi treatment led even to increased phospho-AKT levels. This SARB-specific effect is promising since recent clinical data of Phase 1 and 2 support the potent impact of PI3K/AKT or WNT/B-Catenin inhibitors^51,52^.

Given that WNT signaling pathway and CD133 are interconnected in promoting resistance to conventional therapies^10,41,53–55^, we showed dramatically decreased CD133 level after SARB and Combi treatment. This suggests that SARB might target a broader spectrum of CSC-related players. Indeed the self-renewal capacity was targeted by SARB in two different functional assays, the spheroid formations assay and the propagation assay. In the CAM model a pre-treatment protocol was used and only the resistant tumor cell population was transplanted. If CSCs were eradicated, the tumor cells should have lost the potential of self-renewal and should not grow well on the CAM. Both, Combi and SARB treatment significantly reduced the tumor growth, only small cell aggregates or single cells were visible. The reduction in ß-Catenin levels after SARB treatment was a general effect whereas the reduction of the CD133+ population was cell line specific. Thus, we suggest that SARB has the potential to reduce at least one of these two stemness players. We also cannot exclude treatment effects on other CSC-related markers.

From NanoString analysis, we learned that angiogenesis-associated genes, such as FZD3, MAPK3, AKT3, VEGFA, CTNNB1, and FOS, were significantly downregulated after SARB treatment. These anti-vascular properties of SARB− and partly of Combi were visible in the CAM model. Interestingly 5-FU, led to remarkable bleeding in the grafts, a well-known side effect that could be associated with the spreading of tumor cells^56^. In Combi-treated grafts the tumor environment showed an accumulation of desmoplasia with increased peritumoral lymphoplasmocytic infiltration and fibrosis. Thus, we suggest that SARB might have a lower side-effect spectrum than the Combi or single drug treatments.

This work supports the hypothesis that linking two molecules into a single compound did not simply reflect the Combi-treatment effects, but rather showed targeting of a completely new gene signature. Our findings already give valuable insights into the potential mechanism of action of the novel hybrid drug candidate SARB for CRC.

## Materials and methods

### Chemical evaluation

Detailed information about synthesis of hybrid compounds are given in the supplementary information. ^1^H NMR and ^13^C NMR spectra were recorded at RT on a Bruker Avance spectrometer operating at 300, 500, and 600 MHz. ESI mass spectra were recorded on a Bruker micrOTOF II focus TOF MS-spectrometer. Elemental analysis (C, H, N), carried out with an Euro EA 3000 (Euro Vector) machine and an Elementar vario MICRO cube machine, calculated values confirm a purity of >95%. We obtained the crystals of SARB hybrid in solvent acetonitrile and molecular structure was unambiguously determined by X-ray crystallography (Fig. 1). The data for the crystal structure of SARB hybrid can be found free of charge in: The Cambridge Crystallographic Data Centre (via www.cam.ac.uk/data_request/cif with the deposition number CCDC-1856382).

### Cell lines and reagents

Human colon carcinoma HCT116 and HT29 cells were purchased from ATCC and maintained in RPMI 1640 (PAN Biotech) supplemented with 10% FBS (PAN Biotech) and 1% penicillin and streptomycin (PAN Biotech). Rectal SW837 cells were kindly provided by Dr. Kaulfuß (Institute of Human Genetics in Göttingen) and mantained in the same conditions as HCT116 and HT29. SW620 cells derived from metastatic site, were purchased from ATCC and maintained in DMEM medium (Gibco/Life Tecnologies) supplemented with 10% FBS and 1% penicillin and streptomycin. Normal human intestinal epithelial HCEC cells were grown in basal HCEC medium (PAN Biotech) supplemented with l-glutamine (Gibco/Life Tecnologies), ascorbic acid (Sigma), dexamethasone (Sigma Aldrich), retinoic acid (Sigma Aldrich), and bovine pituitary extract (PromoCell). 5-FU resistant HCT116 cells were obtained from the group of Prof. Lee M. Ellis (MD Anderson, TX, USA) and cultured in minimal essential medium (MEM, Sigma-Aldrich) enriched with 10% FBS, 1% sodium pyruvate 100 mM (Gibco/Life Tecnologies), 1% MEM NEAA non-essential amino acids 100× solution (Gibco/Life Tecnologies), MEM Vitamin solution 100× (Gibco/Life Tecnologies), 1% l-glutamine 200 mM 100x (Gibco/Life Tecnologies), and 1% P/S (Gibco/Life Tecnologies). In order to maintain the resistance, 7.7 µM of 5-FU were added to the cells with each medium change. The cell lines were mycoplasma free and were authenticated using Multiplex Cell Authentication by Multiplexion.

### Chemical compounds

The single compounds, 5-fluorouracil (5-FU) and thymoquinone (TQ) were purchased from Sigma Aldrich. All investigated compounds were dissolved in 100% dimethyl sulfoxide (DMSO) (Pan Biotech) to 50–200 mM stock solutions, and stored at −20 °C. The stock solutions were further diluted in cell culture medium.

### Crystal violet assay

Crystal violet was performed as previously described^26^. Briefly, HCT116, HT29, and HCEC cells were seeded at a density of 7.5 × 10^3^ cells/well in 100 μl of growth medium in 96-well plates (Corning). After 24 h the cells were treated with the compounds at various concentrations for 48 h. IC_50_ values were calculated from at least two biological replicates each performed in at least three technical triplicates.

### Annexin V assay for apoptosis detection

Apoptosis was quantitated by flow cytometry using the Annexin-V-FITC staining kit (Miltenyi), according to the manufacturer´s protocol. Shortly, 5 × 10^5^ HCT116 and HT29 cells were seeded in 6 cm cell culture dishes and treated after 24 h of adherence, with TQ 40, 5-FU 15, Combi 35 + 35, and SARB 35 μM. Floating and adherent cells were harvested after 48 h of treatment by trypsinization. After inactivation of trypsin with 2 ml of growth medium, cells were centrifuged, washed in binding buffer (included in the kit), stained with Annexin-V-staining solution (10 μl Annexin-solution in 100 μl of binding buffer) and incubated for 15 min at RT in the dark. The washing step was repeated, the cells were resuspended in 500 μl binding buffer and stained with 5 μl propidium iodide (PI) directly before analysis with a BD FACSCanto^®^ II flow cytometer (BD Biosciences) and FlowJo software. Unstained cells served as a background reference and untreated cells as negative controls.

### Drug treatment of ex vivo crypt-derived organoids

In order to evaluate the cytotoxicity of our compounds in normal cells, we treated murine intestinal crypt-derived organoids that were grown ex vivo. We used the protocol of Toshiro Sato^57^ with minor variations. The small intestine from 8-week-old C57BL/6J was harvested and washed with a syringe to remove feces. After several washing steps, the SI was opened longitudinally and villi were scraped off using a cover slip. Then the SI was cut into 2–4 mm large fragments and put into PBS. Four to five washing steps with fresh PBS were done to remove any villi or fecal contamination. Afterwards, the fragments were incubated for 15 min at RT with the gentle dissociation solution (STEMCELL Technologies) and crypts were liberated by vigorous shaking. The fragments were removed and the crypts in the supernatant were pelleted. The crypts were resuspended in Matrigel and 20 µl of the crypt–Matrigel suspension were seeded in a 48-well plate. The Matrigel droplet was incubated for 10 min at 37 °C and finally overlaid with 250 µl of IntestiCult™ organoid growth medium (Mouse) (STEMCELL Technlogies). Fresh medium was supplied every 2–3 days and organoids were passaged every 5–7 days. After the first passage, fully developed organoids were liberated from the Matrigel and washed with PBS several times. Organoids were then physically disrupted by pipetting up and down to release single crypts. Single crypts were then equally resuspended in 50% Matrigel and 10 µl of the suspension were seeded into pre-warmed 96-well plates. After solidifying of the Matrigel, the crypt droplets were overlaid with 100 µl IntestiCult™ organoid growth medium. Three days after seeding (passage 2) the organoids were treated (passage 2, day 3–5 after seeding) with the corresponding drugs; TQ 40, 5-FU 15, Combi 35, SARB 35, and SARB 50 µM for 48 h. The viability of the organoids was assessed by CellTiter-Glo^®^ 3D Cell Viability Assay (#G9682, Promega) according to the manufacturer’s instructions.

### Collection of the cell pellets for RT-qPCR and NanoString analysis

HCT116 cells were used for NanoString analysis and RT-qPCR. The cells were treated at 50–60% confluency with TQ 40, 5-FU 15, Combi 35, and SARB 35 µM for 24 h. The collection of cell pellet was performed on ice and the cells were scraped from the plates and the respective supernatants with the cells were transferred to a 50-ml tube, washed with ice cold PBS and the remaining cells were scraped and centrifuged for 5 min, 5000 rpm at 4 °C. The supernatants were then discarded and the cells were washed with 1 ml cold PBS, transferred to 1.5 ml tube and centrifuged another time with the same settings. The supernatants were then discarded, the pellets were frozen in liquid nitrogen and stored at −80 °C.

### RT-qPCR analysis

RNA was isolated using QIAzol^®^ Lysis Reagent (Qiagen) in combination with RNeasy Mini Kit (Qiagen) according to the manufacturer’s protocols. cDNA synthesis was performed with the QuantiTect Reverse Transcription Kit (Qiagen) according to the manufacturer. CFX96TM Real-Time System (Bio-Rad) and the C1000^TM^ Thermal Cycler (Bio-Rad) were used to measure the Ct values for investigated genes which were normalized to human B2M expression. The primers used in this study are given in Suppl. Table S4.

### NanoString gene expression analysis

The NanoString PanCancer Pathway panel gene expression assay was performed according to the manufacturer´s instructions with 50 ng of total RNA. Data processing was performed using nSolver Analysis Software 3.0 (NanoString Technologies). All samples were normalized using the geometric mean of the housekeeping genes. Differentially expressed genes between the treated and the untreated control cells were given as fold change expression. Deregulated KEGG pathways were identified via STRING software (http://string-db.org). The Gene Expression Omnibus (GEO) database (GSE34053 dataset) was used to identify the expression profile of key genes in CD133± colon cancer populations. The GEO2R web tool was used to identify differentially expressed genes. NanoString results are deposited at GEO and GSE122860 accession number provides access to the raw and processed data.

### Western blot analysis

Western blot analysis was performed as previously described^38^. Briefly, 30 or 40 μg of total protein were separated by 10% denaturing SDS–PAGE and transferred onto 0.45 μm nitrocellulose membranes overnight. The membranes were incubated with primary ß-Catenin (6B3) (1:1000, Cell Signaling, #9582), CD133 (1:250, Miltenyi, #130-092-395), p-AKT (Ser 473) (D9E) XP (1:1000, Cell Signaling, #4060), AKT (1:1000, Cell Signaling, #9272), p-MEK1/2 (Ser217/221) (1:1000, Cell Signaling, #9121), or MEK1/2 (1:1000, Cell Signaling, #9122) antibodies overnight at 4 °C. Secondary horseradish-peroxidase-coupled antibodies goat-anti-mouse IgG (H + L) (1:10,000,Thermo Fisher Scientific) or anti-rabbit IgG (H + L) (1:10,000, Thermo Fisher Scientific), and anti-biotin, HRP-linked antibody (1:5000, Cell Signaling) were then applied for 1 h at RT. Hybridization with GAPDH-HRP (6C5) (1:10,000–20,000, Abnova, #MAB5476) coupled antibody was performed for 30 min at RT as a housekeeping gene. Antibodies were visualized using the Immobilon Western Chemiluminescent horseradish peroxidase (HRP) substrate kit (Merck Millipore). Images were generated using Gene Genome Syngene Bio Imaging and quantified using Image J (Rasband, W.S., U.S. National Institutes of Health).

### TOP/FOP-FLASH Luciferase Reporter assay

HCT116 cells (20 × 10^3^) were co-transfected for 6 h before 24 h treatment with 10, 20, 50 µM SARB hybrid either with 100 ng Super8xTOP-FLASH or 100 ng Super8xFOP-FLASH, respectively, and 10 ng ß-galactosidase reporter plasmid for normalization of protein amounts. Plasmids were kindly provided by Prof. Trevor Dale, Cardiff University School of Biosciences. The reporter activity was assessed by adding 50 µl of Beta-Glo (#E4720, Promega) or 50 µl of Bright-Glo (#E2610, Promega) to the 50 µl of lysed samples, following the manufacturer instructions and measured by a luminometer (Victor^TM^ X3 Multilabel reader). The results were normalized to total protein amounts and were expressed relative to DMSO controls.

### Flow cytometry analysis of CD133

HT29 and HCT116 cells were treated at 50–60% confluence for 48 h with Combi 35 µM, SARB 35 µM or were left untreated. After trypsinization the cells were collected in 100 µL of buffer solution containing PBS, 0.5% BSA, and 2 mM EDTA by diluting MACS BSA Stock Solution (# 130‑091‑376, 1:20) with auto MACS rinsing solution (#130‑091‑222) and were incubated according to the manufacturer´s suggestion with mouse anti-human CD133/1 (AC133) conjugated with VioBright FITC (#130-105-226, 1:11; Miltenyi Biotec), or the IgG isotype control after blocking the Fc receptors with the FcR-blocking reagent (# 130‑059-901). The analysis was made with a BD FACSCanto^®^ II flow cytometer (BD Biosciences) and FlowJo software. All events were included in the analysis.

### Wound healing assay

HCT116 cells were seeded in culture plates (six-well plates) and allowed to adhere for minimum one day in the respective medium. The scratch was done in three different area of the well when the cells were completely confluent, using a sterile 200 µl tip and the plates were washed with PBS. The medium alone or with the compounds (Combi 35 µM or SARB 35 µM) was added to the cells and the plates were monitored immediately after the treatment, corresponding to 0 h, as well as at 24 and 48 h. The data is presented as the percentage of the wound closure which was calculated: (wound length at 0 h)−(wound length at 24 or 48 h)/(wound length at 0 h) × 100. The data are calculated from three independent experiments, each done in duplicate.

### Spheroid formation assay (SFA)

To investigate the capacity of CRC cells to grow under non-adherent conditions, cells were seeded at a density of 1.5 × 10^6^ cells in 10 cm plates. After 24 h of recovery, the cells were treated with increasing concentrations of SARB (10, 20, 50 μM) or Combi 35 µM. Incubation continued for another 48 h, then the cells were washed with PBS, trypsinized and 5 × 10^4^ cells were then seeded in ultra-low attachment (ULA) six-well plates (Corning) in 2 ml of CSC medium (PromoCell) and 40 μl Matrigel with growth factors (Corning). Spheroid formation was monitored 7 days after seeding and then the spheroids were transferred to 15 ml falcon tubes, washed with PBS, trypsinzed, and 2.5 × 10^4^ cells were seeded in ULA six-well plates with 2 ml CSC medium and 40 μl Matrigel. Cells were let grow for another 14 days and fresh medium was added every 2–3 days. At day 21, the spheroid growth was documented by light microscopy using a Nikon Eclipse Ti–S microscope or Leica DMI1 light microscope. The images were then analyzed using a macro (given in SI) which was imported to ImageJ (modified from ref. ^58^) to determine spheroid number.

### Propagation assay

For the propagation assay, single cell suspensions of HCT116 cells were suspended in Matrigel (Growth Factor Reduced) and in serum-free RPMI medium (1:1) at a concentration of 2000 cells/well in a total volume of 50 µl. The solution was plated in 24-well plate and was allowed to solidify for 1 h at 37 °C. The RPMI medium supplemented with 5% FBS and 1% P/S without or with the drug at the indicated concentrations was added in triplicate. The medium containing the drug was refreshed every 2–3 days. The spheres were counted and harvested between 9 and 13 days after plating (generation 1). For the propagation, the spheres were collected and the Matrigel was digested by incubation in 500 µl of serum-free media containing dispase (1 mg/ml) (Corning) for 1 h at 37 °C. The resulting sphere containing solution was collected, centrifuged and the resulting pellet was re-suspended in Trypsin (5–10 min) and later inactivated by adding RPMI media (5% FBS). The cells were than centrifuged, washed, suspended in serum-free RPMI and the number of viable cells was determined using trypan blue. Cells were again suspended in Matrigel-medium mixture and were plated as described above. Spheres were propagated up to five generations and the number of spheres was calculated for each generation using the same macro as for the SFA.

### Generation of spheroid-derived CD133 enriched sub-clones

We used HCT116 cells which were seeded into 30–35% Matrigel (growth factor reduced, Corning) in RPMI medium (supplemented with 10% FBS + 1% P/S; 1× volume Matrigel + 2× volume medium). A total volume of 2 × 10^3^ single cells/well was seeded into one well of a six-well plate, incubated at 37 °C for 15 min until the Matrigel solidified and covered with 300–500 µl medium. The medium was changed every 2–3 days and after 7 days of incubation, single cell colonies were picked gently with a tip of a sterile toothpick. The individual colony was then transferred into one well of a 96-well plate, which was filled with 100 µl RPMI medium beforehand. The cells were cultivated until a colony started to grow and only wells with single colonies were used. Seven cell lines (ST1–ST7) were generated from these clones, which were then used for further experiments. The generated clones were monitored by microscopic images (Leica DMi1, Leica Microsystems).

### Immunofluorescence staining

2D HCT116 and HT29 cells were grown (8 × 10^4^ cells/well) in eight-well chambered coverslips (Ibidi) and treated after 2 days of adherence for 48 h with increasing concentrations of SARB (10, 20, 50 µM). Spheroids (3D) at day 21 derived from SFA and 2D cells were washed with PBS, fixed with 4% paraformaldehyde for 20 min at RT, permeabilized with 0.1% TritonX in PBS (15 min at RT), blocked for 30 min with 3% BSA (w/v) in PBS, and stained with a 1:250 dilution of primary antibody against CD133 (Miltenyi Biotec) or 1:100 of ß-Catenin (D10A8) XP (Cell Signaling) overnight at 4 °C. The next day, after a washing step, the spheroids stained with CD133 were incubated with a 1:250 goat anti-mouse dilution of the secondary antibody (Alexa Fluor 555, Invitrogen), where the cells labeled with ß-Catenin were incubated with 1:100 goat anti-rabbit IgG (Alexa Fluor 555, Invitrogen) for 1 h RT, washed again, stained with 1:1000 Hoechst 33342 (Sigma) in PBS (1 mg/ml stock solution) per well and imaged with a Nikon Eclipse Ti–S.

### Chorioallantoic membrane (CAM) assay and immunohistochemical staining

The chick embryo CAM xenograft assay was performed as previously described^37,38^. Shortly, 1 × 10^6^ HCT116 cells were pretreated with the compounds and were applied in a 1:1 medium-Matrigel mixture (Corning) onto the CAM. After 5 days of incubation the generated micro-tumors were harvested. The CAM tumors were photographed and the tumor volume was calculated using the formula: *V* = (*L* × *W* × *H*)/2 (*n* ≥ 9 for all groups). The CAM microtumours were then fixed in 4% phosphate buffered formalin for 24 h, dehydrated and embedded in paraffin. The HE staining and the scanning of the slides were done as previously described^38^. The percentage of vital tumor cell areas and mitosis was evaluated (*n* = 7 control-CAM, *n* = 7 TQ-CAM, *n* = 7 5 FU-CAM, *n* = 8 Combi-CAM, and *n* = 8 SARB-CAM microtumors).

The rate of mitotic cells was determined using HE-stained sections of the CAM micro-tumors and the high-power-field (HPF) method. Immunohistochemical labeling for β-Catenin (1:50, BD Bioscences), E-cadherin (1:2000, BD Bioscences), and Ki67 (1:100, Dako) was performed according to standard routine procedures for immunohistochemical analysis.

### Statistical analysis

All statistical analyses (*t*-test and one-way ANOVA) were performed using GraphPad Prism 7 (version 7.0, GraphPad Software Inc., La Jolla, CA, USA).

## Supplementary information

Suppl. Information

Figure S1

Figure S2

Figure S3

Figure S4

Figure S5

Figure S6
