# Supplementary material for: Combination of 5-fluorouracil and thymoquinone targets stem cell gene signature in colorectal cancer cells
Source: Cell Death Dis. 2019 May 16;10(6):379. doi: 10.1038/s41419-019-1611-4 (PMC6522523; doi:10.1038/s41419-019-1611-4)
Supplement: Supplementary file 1 — Suppl. Information [file 41419_2019_1611_MOESM1_ESM.docx]

**Supporting Information**

**Combination of 5-Fluorouracil and Thymoquinone targets stem cell gene signature in colorectal cancer cells**

# Benardina Ndreshkjana^1,2*^, Aysun Çapcı^3*^, Volker Klein^3^, Pithi Chanvorachote^4^, Julienne K. Muenzner^1,2^, Kerstin Huebner^1,2^, Sara Steinmann^1,2^, Katharina Erlenbach-Wünsch^2^, Carol I. Geppert^2^, Abbas Agaimy^2^, Farah Ballout^5^, Chirine El-Baba^1^, Hala Gali-Muhtasib^5^, Adriana Vial Roehe^6^, Arndt Hartmann^1,2^, Svetlana B. Tsogoeva^3^, Regine Schneider-Stock^1,2^

^1^University Hospital, Friedrich-Alexander-University Erlangen-Nuremberg, Experimental Tumorpathology, Institute of Pathology, Erlangen, Germany; ^2^Institute of Pathology, Friedrich-Alexander University of Erlangen-Nuremberg, Erlangen, Germany; ^3^Organic Chemistry Chair I and Interdisciplinary Center for Molecular Materials (ICMM), Friedrich-Alexander University Erlangen-Nuremberg, Erlangen, Germany; ^4^Department of Pharmacology and Physiology, Faculty of Pharmaceutical Sciences, Chulalongkorn University, Bangkok, Thailand; ^5^Department of Biology and Center for Drug Discovery, American University of Beirut, Beirut, Lebanon; ^6^Department of Pathology, Federal University of Health Sciences of Porto Alegre (UFCSPA), Porto Alegre, Brazil

**Table of Contents:**

*Experimental chemical section S3-S8*

*Recorded spectra S9-S14*

*Tables S15-S17*

*Macro protocol S18-S19*

*Western Blot S20*

*References S20*

*Figure legends S21-S23*

**Experimental section**

**Synthesis of thymoquinone derivative 5**

In a 25 mL two-neck flask, thymoquinone (**1**) (0.61 mmol, 100 mg, 1.0 eq) and succinic acid (0.49 mmol, 58 mg, 0.8 eq) were dissolved in mixture of acetonitrile (3 mL) and water (3 mL) under N_2_ atmosphere. Then AgNO_3_ (0.061mmol, 10 mg, 0.1 eq) was added. The reaction mixture was heated up to 80^°^ C and (NH_4_)_2_S_2_O_8_ (0.61 mmol, 139 mg, 1.0 eq) was added drop wise. The reaction mixture was refluxed at 100^°^ C overnight and then it was cooled to room temperature (RT) and diluted with H_2_O (0.6 mL). The mixture was extracted with Et_2_O (5x5 mL), organic phases were combined and dried over Na_2_SO_4_ and evaporated at atmospheric pressure. The crude product **5** was purified as yellow oil by column chromatography in 45% yield Rf: 0.56 (SiO_2_, cyclohexane/EtOAc/aceton 6:4:0.5, UV and phosphomolybdic acid in ethanol 10%). Anal. calcd. for C_13_H_16_O_4_: C, 66.09, H, 6.83, found: C, 66.15, H, 7.03. The spectroscopic data were verified in accordance with literature**^1^**.

**Synthesis of 5-FU derivative 6 and the hybrid compound SARB**

In an evacuated Schlenk tube 5-FU (**2**) (1.31 mmol, 175 mg, 2.0 eq) was dissolved in formaldehyde 37% (0.8 M, 1.65 mL) at 60^°^ C, under N_2_ atmosphere. The reaction mixture was stirred for 45 min. Afterwards solvent was evaporated under reduced pressure. The crude product **6** was dried overnight in high vacuum (up to 10^-3^) and directly used without further purification**^2^**. The white oily compound **6** was dissolved in acetonitrile (0.45 mL). Then DCC (0.73 mmol, 150 mg, 1.1 eq) and DMAP (0.033 mmol, 4 mg, 0.05 eq) were added at -10^°^ C. To this solution, thymoquinone derivative **5** which was dissolved in acetonitrile (1 mL) was added drop wise. The mixture kept at -10-0^°^ C for 1 h and it was stirred overnight at RT. The urea was filtered and washed with EtOAc. The solvent was evaporated under reduced pressure and the crude product was purified via column chromatography. Hybrid compound (SARB) was obtained as a yellow solid (36%). Rf: 0.38 (SiO_2_, cyclohexan/EtOAc/aceton 6:4:0.5, UV and phosphomolybdic acid in ethanol 10%).

^1^H-NMR (400 MHz, CDCl_3_): δ = 1.10 (d, *J*= 6.9 Hz, 6 H), 2.03 (s, 3 H), 2.54 (m, 2 H), 2.83 (m, 2 H), 3.02 (dsept, *J* = 6.9, 1.1 Hz, 1 H), 5.62 (s, 2H), 6.49 (d, *J*= 1.1 Hz, 1 H), 7.61 (d, 1H), 8.44 (s, NH) ppm.

^13^C NMR (100 MHz, CDCl_3_): 187.8, 186.7, 172.5, 156.5, 154.6, 148.8, 142.2, 141.4, 138.9, 130.2, 128.7, 69.8, 32.4, 26.7, 22.1, 21.3 (2x), 11.8 ppm.

^19^F NMR (300 MHz, CDCl_3_): -164.03-163.99 (t, 1F) ppm.

IR (ATR): 3195, 3077, 2964, 2932, 2873, 1708, 1645, 1457, 1361, 1248, 1128, 1050, 977, 889, 781, 709, 549, 415.

MS (MALDI) m/z: 401 ([M+Na)]^+^); HRMS (ESI) m/z calculated for [C_18_H_19_FN_2_NaO_6_]^+^: 401.11248, found: 401.11178.

Anal. calcd. for C_18_H_19_FN_2_O_6_: C, 57.14, H, 5.06, N, 7.40, found: C, 57.15, H, 4.92, N, 7.24.

**Synthesis of thymoquinone derivative 3**

In a 50 mL two-neck flask, thymoquinone (**1**) (0.61 mmol, 100 mg, 1.0 eq) and sodium azide (0.78 mmol, 51 mg, 1.3 eq) were dissolved in ethanol (3 mL) under N_2_ atmosphere. The glacial acid (1 mL) was added dropwise and the reaction mixture was heated to reflux for 3 h. Afterwards, the reaction mixture was cooled down to RT, diluted with H_2_O and extracted with Et_2_O. The organic phase was dried over Na_2_SO_4_ and removed under reduced pressure. The crude product was purified by column chromatography (SiO_2_, hexane/EtOAc 4:1 > 2:1). The product **3** was obtained as red oil (48 mg, 0.27 mmol, 44% yield). The spectroscopic data were verified in accordance with literature**^3^**.

**Synthesis of 5-fluorouracil-1-acetylchloride (4) and the hybrid compound AC29**

In a 25 mL two-neck flask KOH (195 mg, 3.5 mmol, 4.5 eq) and 5-FU (**2**) (100 mg, 0.77 mmol, 1.0 eq) were dissolved in water (10 mL). The reaction mixture was heated up to 40^°^ C, and then the solution of bromoacetic acid (194 mg, 1.4 mmol, 1.8 eq) in water (0.4 mL) was added. The reaction mixture was heated up to 80^°^ C and was stirred for 5 h while keeping the pH of the solution at 10 by KOH solution. Then the reaction mixture was cooled to RT and HCl was added to adjust pH to 2 and then solvent evaporated under reduced pressure. The crude product was dissolved in aqueous solution of NaHCO_3_ and was recrystallized by HCl. The white crystalline 5-fluorouracil-1-acetic acid was obtained in quantitative yield. The spectroscopic data were verified in accordance with literature**^2^**. Following, 5-fluorouracil-1-acetic acid and thionyl chloride (1 mL) were mixed in the presence of 1 drop of DMF at 0^o^ C, under N_2_ atmosphere. The reaction mixture was refluxed for 5 h and excess of SOCl_2_ was removed under vacuum. The crude product **4** was used without further purification**^2^**. Afterwards, the 5-fluorouracil-1-acetyl chloride (**4**) was dissolved in DMF and previously prepared thymoquinone derivative **3** was added to the solution. The reaction mixture was stirred overnight. The consumption of thymoquinone derivative indicated the completion of the reaction. The solvent removed under reduced pressure and the crude compound purified via column chromatography Rf: 0.29 (SiO_2_, hexane/EtOAc/MeOH 6:4:0.5, UV and phosphomolybdic acid in ethanol 10%). Hybrid AC29 was obtained as yellow solid (30 mg, 0.085 mmol, 32%)

^1^H-NMR (500 MHz, DMSO-d_6_): δ = 1.08 (d, *J*= 6.9 Hz, 6 H), 1.77 (s, 3 H), 2.90 (dsept, 1 H), 4.56 (s, 2 H), 6.61 (d, *J* = 6.1 Hz, 1 H), 8.09 (d, *J* = 6.7 Hz, 1H), 10.06 (s, 1NH), 11.89 (s, 1NH) ppm.

^13^C NMR (126 MHz, DMSO-d_6_): 187.48, 182.69, 165.30, 157.53, 152.86, 149.69, 139.29, 137.01, 134.66, 130.94, 130.42, 50.15, 26.47, 21.04 (2x), 12.41 ppm.

^19^F NMR (470 MHz, DMSO-d_6_): -170.42-170.41 (d, *J*= 6.9 Hz 1F) ppm.

HRMS (ESI) m/z calculated for [C_16_H_16_FN_3_NaO_5_]^+^: 372.09662, found: 372.09675.

Anal. calcd. for C_16_H_16_FN_3_O_5_: C, 55.01, H, 4.62, N, 12.03, found: C, 54.56, H, 4.64, N, 11.79.

**Synthesis of 5-Fluorouracil derivative 8**

5-Fluorouracil (100 mg, 0.77 mmol, 1.00 eq) was dissolved in dry 1, 2-dichloro ethane (4 mL) under N_2_ atmosphere. Trimethylsilyl trifluoromethanesulfonate (277.8 µL, 1.54 mmol, 2.0 eq) then triethyl amine (213.1 µL, 1.54 mmol, 2.0 eq) were slowly added at RT. After 30 minutes, a solution has formed and 80% propargyl bromide solution in toluene (148.6 mg, 111.3 µL, 999.4 mmol) was added. The mixture was allowed to stir overnight. Ice water (40 mL) was added and extracted with EtOAc (3 x 10 mL), dried over MgSO_4_ and purified via column chromatography (SiO_2_, DCM/MeOH 100:0.05). The product **8** was obtained as white solid (56 mg, 333 mmol, 43% yield.) The spectroscopic data were verified in accordance with literature**^4^**.

**Synthesis of thymoquinone derivative 7 and the hybrid compound KV98**

3-(3-hydroxypropyl)-5-isopropyl-2-methylcyclohexa-2,5-diene-1,4-dione**^5^** (55 mg, 0.25 mmol 1.0 eq) and 2-azido-1,3-dimethylimidazolinium hexafluorophosphate**^6^** was dissolved in dry THF (4 mL) under N_2_´-atmosphere and cooled to 0° C. DBU (110.8 µL, 0.75 mmol, 3.0 eq) was added dropwise for 30 min. The reaction was allowed to stir for 2.5 h. Saturated aqueous ammonium chloride solution (15 mL) and DCM (15 mL) were added. The organic phase was collected and the aqueous phase was extracted with DCM (2 x 10 mL), dried over MgSO_4_ and purified via short column chromatography (SiO_2_, hexane/ EtOAc 8:1). The product **7** was obtained as orange/brown solid (30 mg, 0.12 mmol, 49% yield) and directly used in the click reaction without characterization. The compound **7** (27.0 mg, 198 mmol, 1.0 eq), compound **8** (22.0 mg, 131 mmol, 1.20 eq), CuI (1.04 mg, 5.46 mmol, 0.05 eq), acetic acid (0.66 µL, 10.9 mmol, 0.1 eq) and DIPEA (1.86 µL, 10.9 mmol, 0.1 eq) **^7^** were added to 8 mL DCM under N_2_ atmosphere. The mixture was stirred overnight at RT. After 18 h, the reaction mixture was heated to reflux for 3 h until completion of the reaction. The crude was purified via column chromatography (SiO_2_, DCM/Acetone 3:1). The isolated product was recrystallized from DCM to obtain a pure light brown product (30.3 mg, 73.2 mmol, 67% yield)

^1^H NMR (600 MHz, CDCl_3_) *δ* = 8.48 (s, 1H), 7.74 (s, 1H), 7.60 (d, *J* = 5.4 Hz, 1H), 6.48 (d, *J* = 1.2 Hz, 1H), 4.95 (s, 2H), 4.39 (t, *J* = 7.1 Hz, 2H), 3.00 (heptd, *J* = 6.7, 1.0 Hz, 1H), 2.54 – 2.43 (m, 2H), 2.10 – 1.99 (m, 2H), 1.95 (s, 3H), 1.09 (d, *J* = 6.9 Hz, 6H).

^13^C NMR (151 MHz, CDCl_3_) *δ* = 187.8, 186.8, 156.7, 156.5, 154.6, 149.1, 142.8, 141.4, 141.3, 141.0, 139.7, 130.3, 128.6, 128.3, 123.6, 50.3, 43.2, 28.8, 26.8, 23.7, 21.4, 11.8 ppm.

^19^F[^1^H] NMR (470 MHz, CDCl_3_) *δ* = -165 ppm.

HRMS (APPI): *m/z* calculated for C_20_H_23_FN_5_O_4_ [M+H]^+^ 416.1729, found 416.1734.

Anal. calcd. for C_20_H_22_FN_5_O_4_: C, 57.83, H, 5.34, N, 16.86, found C, 57.61, H, 5.56, N, 16.57.

**Recorded spectra:**

^1^H NMR spectrum of hybrid **SARB** recorded on a Bruker Avance spectrometer (300 MHz, CDCl_3_)

^
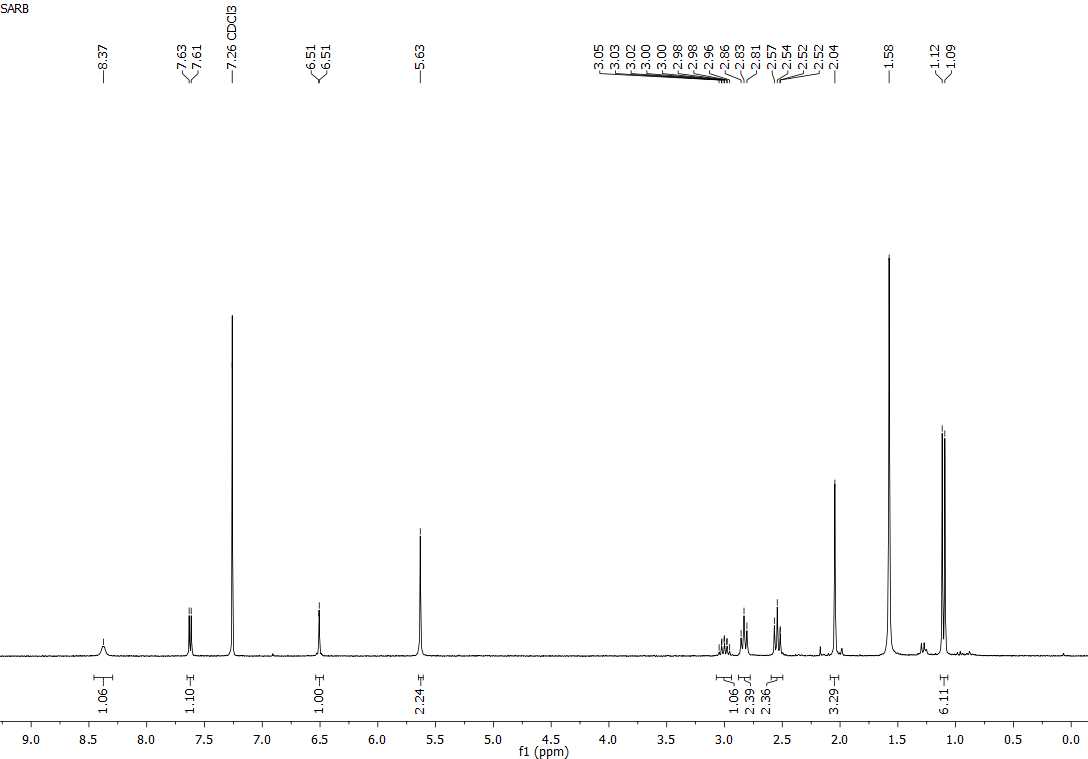
^

^13^C NMR spectrum of hybrid **SARB** recorded on a Bruker Avance spectrometer (75 MHz, CDCl_3_)

^
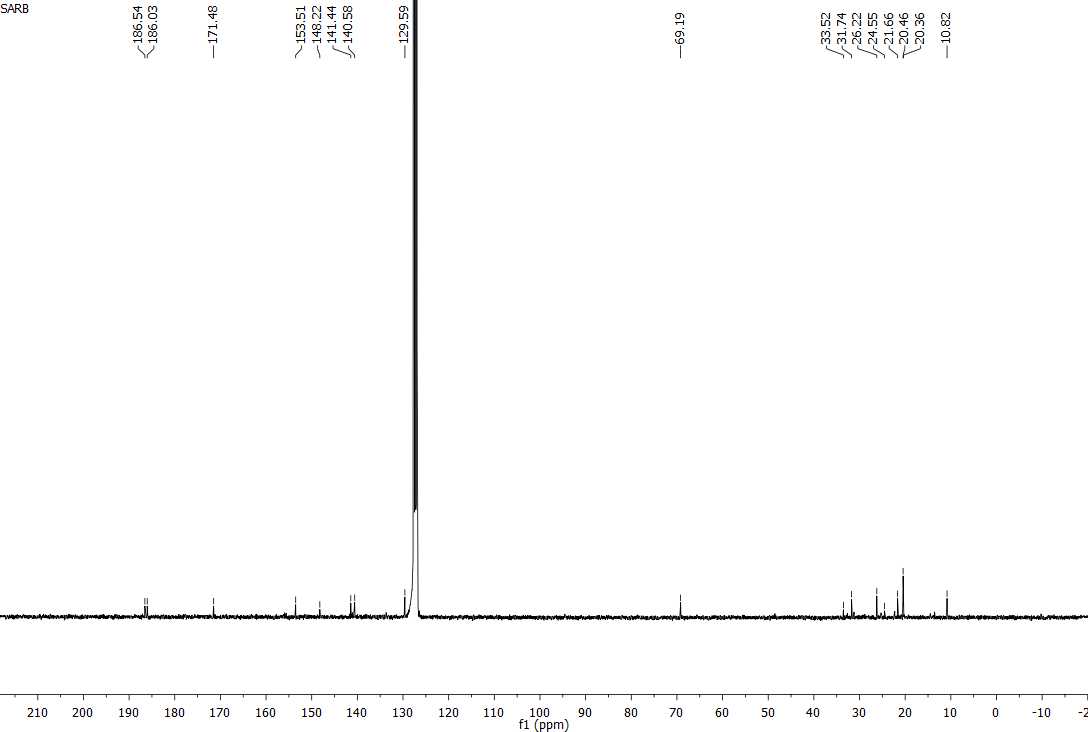
^

^19^F NMR spectrum of hybrid **SARB** recorded on a Bruker Avance spectrometer (470 MHz, DMSO-d_6_)


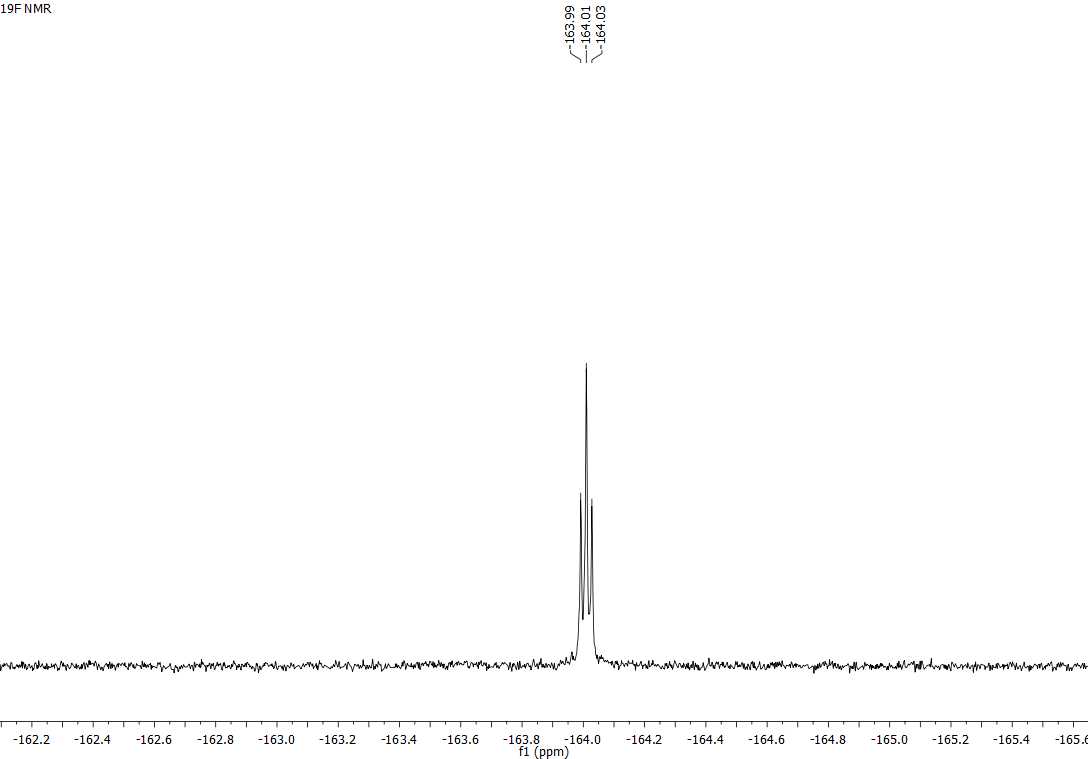


ESI mass spectrum of hybrid **SARB** recorded on a Bruker micrOTOF II focus TOF MS-spectrometer

^
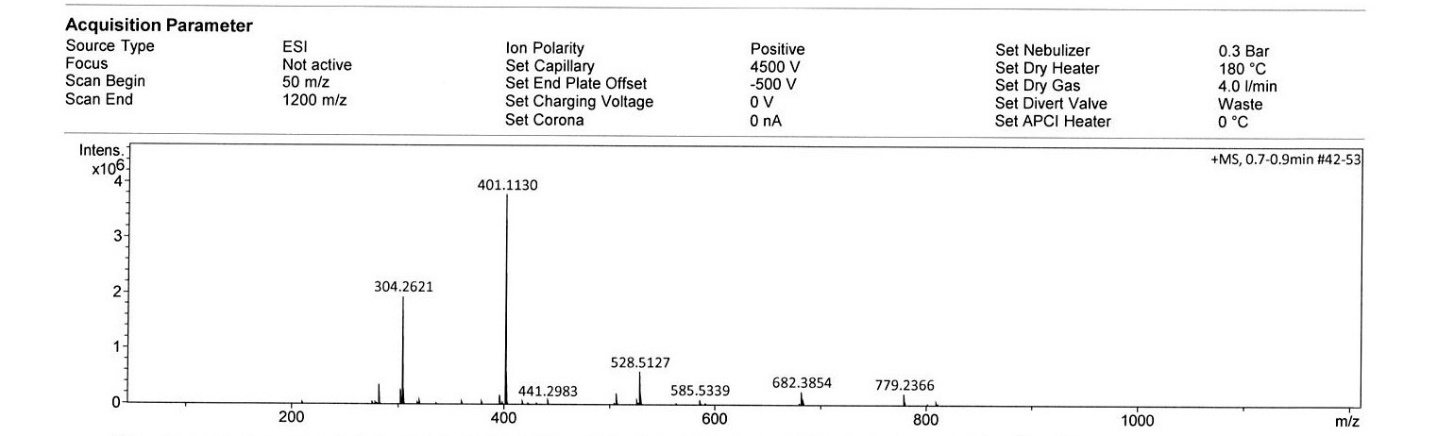
^

^1^H NMR spectrum of hybrid **AC29** recorded on a Bruker Avance spectrometer (500 MHz, DMSO-d_6_)


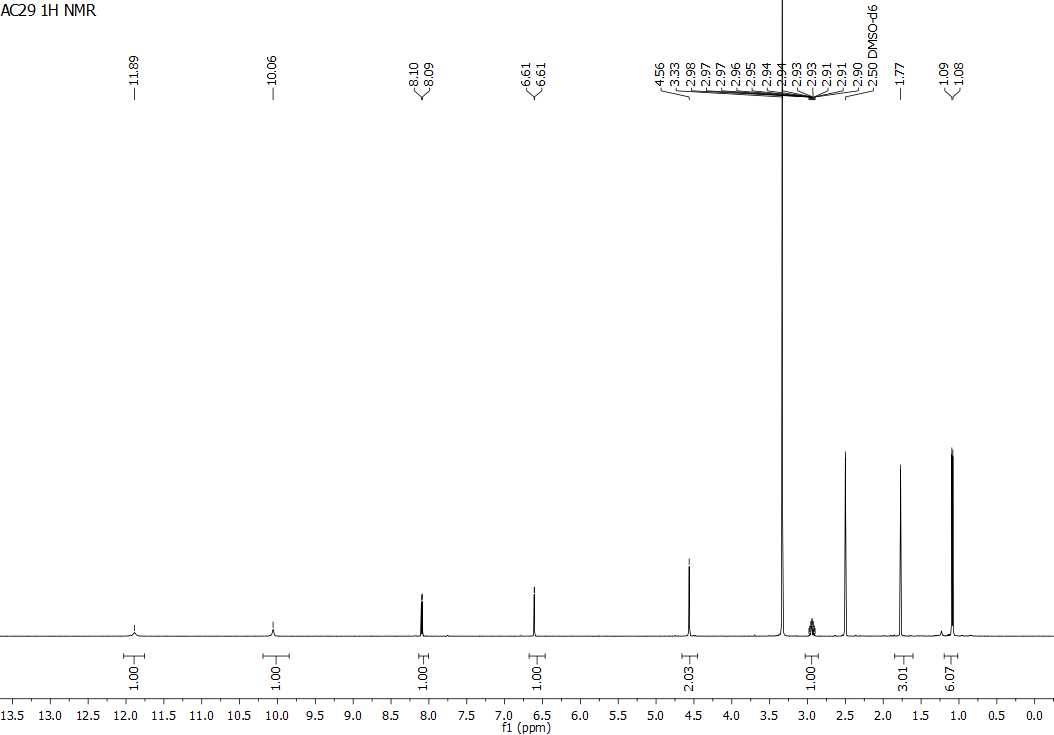


^13^C NMR spectrum of hybrid **AC29** recorded on a Bruker Avance spectrometer (126 MHz, DMSO-d_6_)


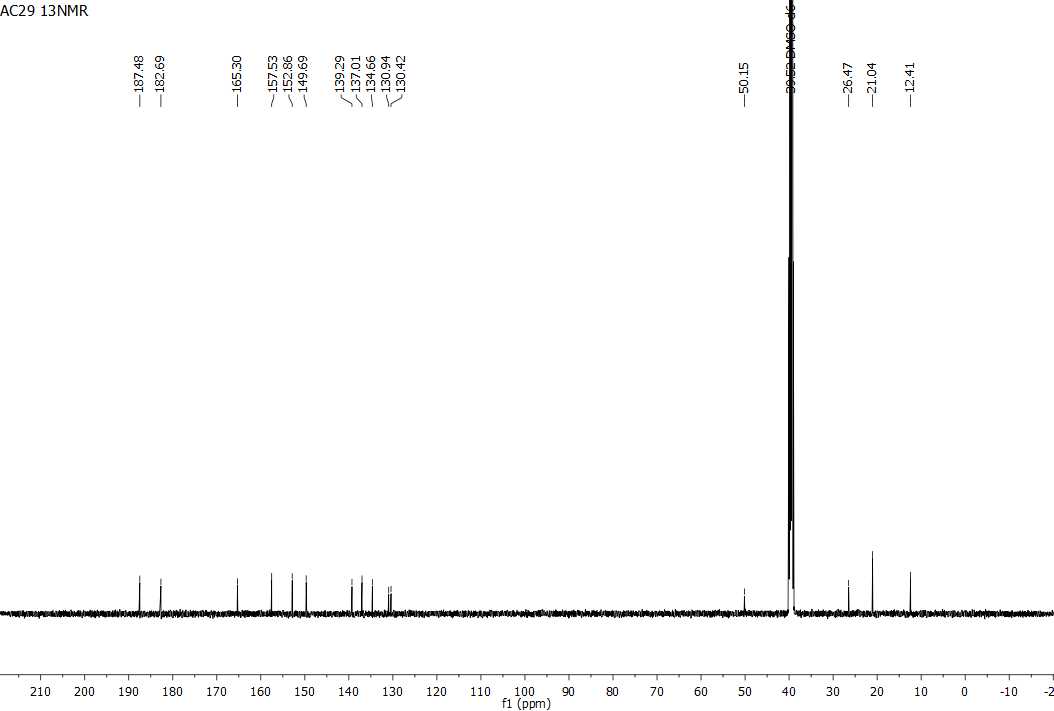


^19^F NMR spectrum of hybrid **AC29** recorded on a Bruker Avance spectrometer (470 MHz, DMSO-d_6_)


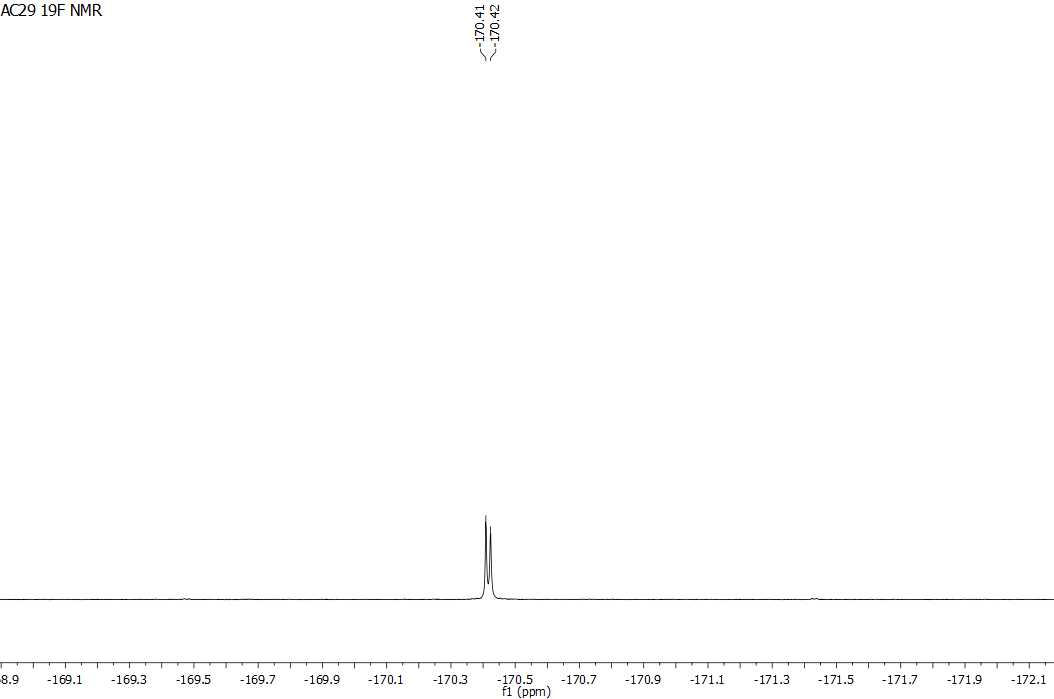


ESI mass spectrum of hybrid **AC29** recorded on a Bruker micrOTOF II focus TOF MS-spectrometer


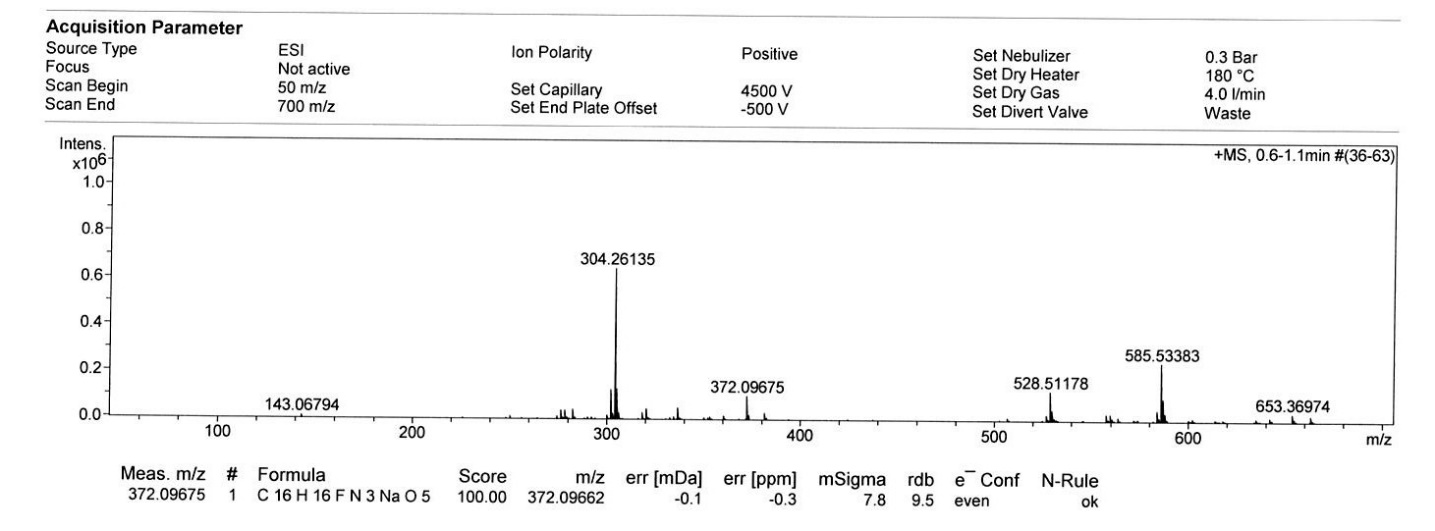


^1^H NMR spectrum of hybrid **KV98** recorded on a Bruker Avance spectrometer (600 MHz, CDCl_3_)


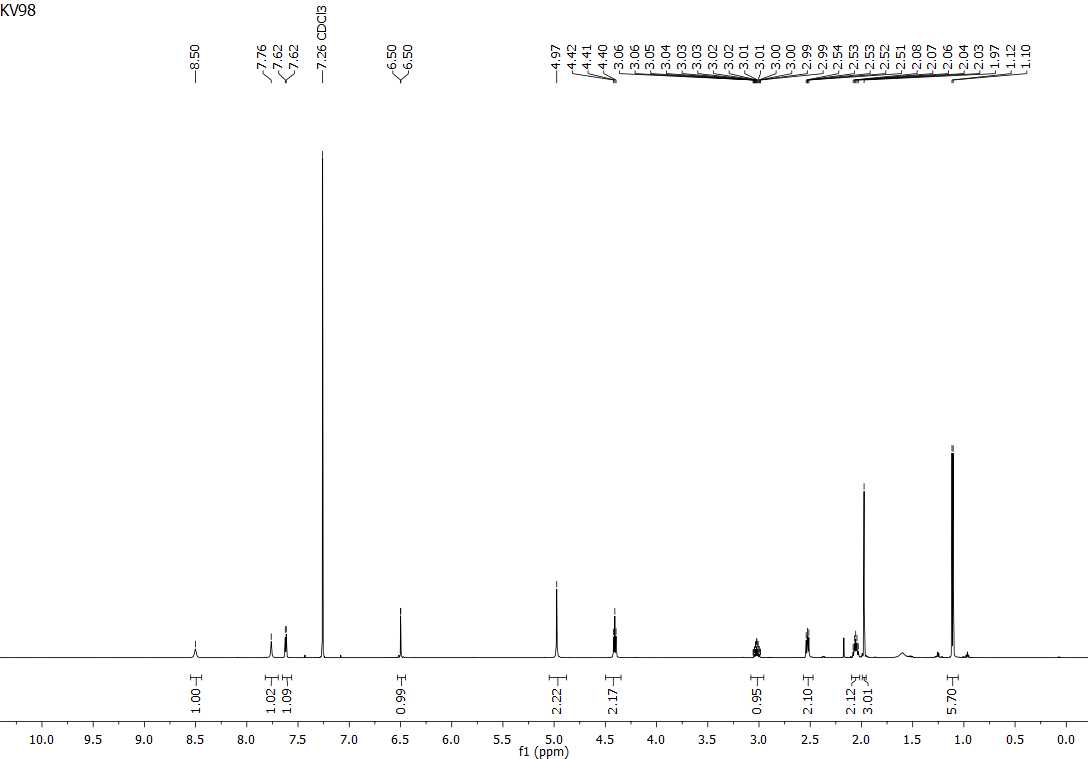


^13^C NMR spectrum of hybrid **KV98** recorded on a Bruker Avance spectrometer (151 MHz, CDCl_3_)


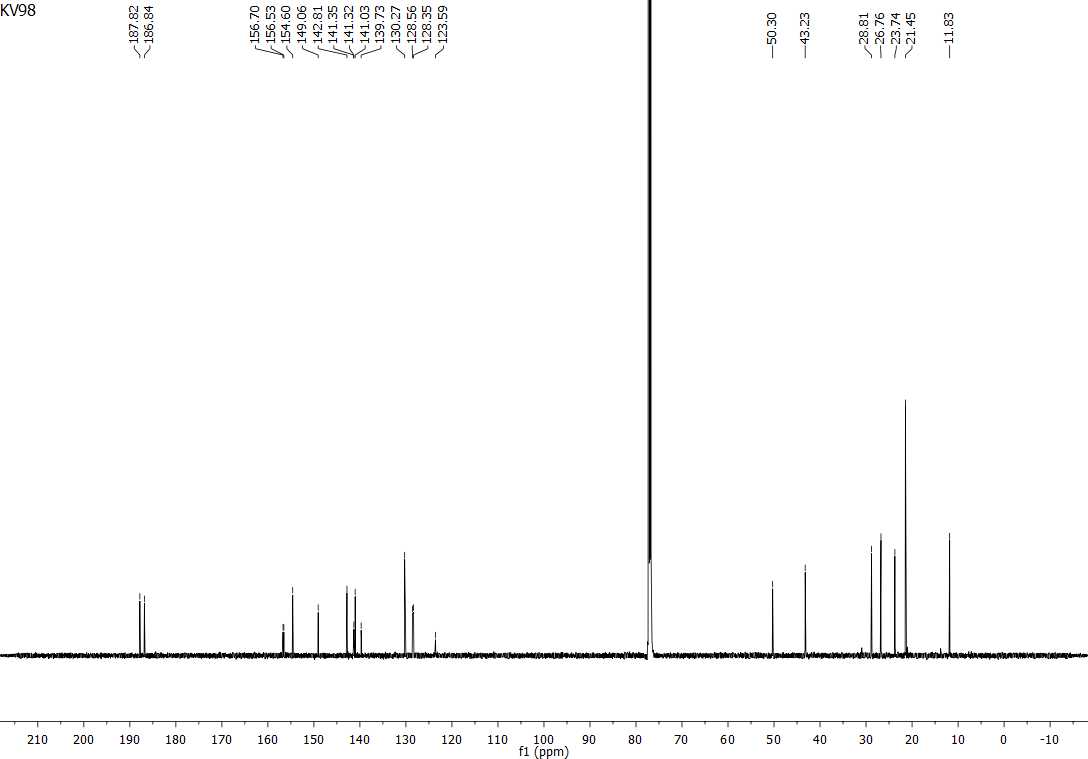


^19^F NMR spectrum of hybrid **KV98** recorded on a Bruker Avance spectrometer (470 MHz, DMSO-d_6_)


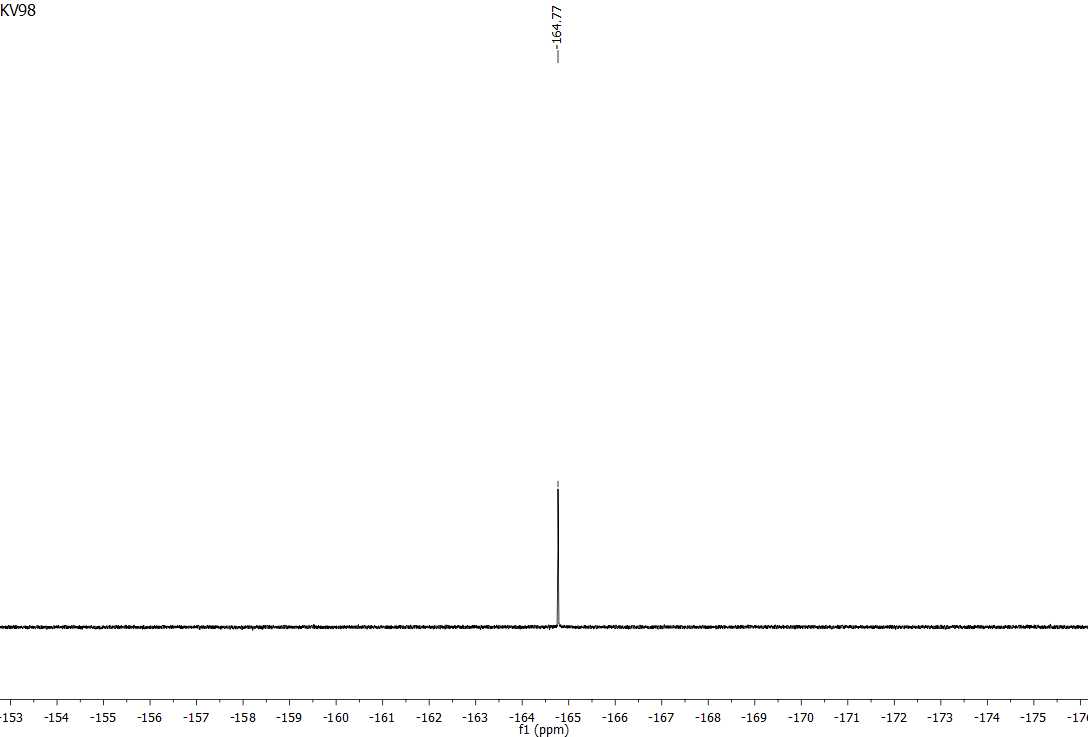


**Material and methods**

**Table S1.** Differentially expressed genes in Combi as compared to the individual treatments.

| Gene Name | Accession # | 5-FU | | p-value | | Combi | p-value | | TQ | p-value |
| --- | --- | --- | --- | --- | --- | --- | --- | --- | --- | --- |
| NGFR | NM_002507.1 | | 5,2 | | 0,00 | 6,8 | 0,00 | 1,1 | | 0,37 |
| NPM1 | NM_002520.5 | | -2,7 | | 0,00 | -3,4 | 0,03 | -1,0 | | 0,95 |
| HIST1H3H | NM_003536.2 | | -10,5 | | 0,00 | -11,9 | 0,00 | -1,2 | | 0,33 |
| JUN | NM_002228.3 | | 2,8 | | 0,00 | 4,2 | 0,00 | 1,4 | | 0,10 |
| FST | NM_006350.2 | | -1,4 | | 0,01 | 3,4 | 0,02 | -1,2 | | 0,72 |
| EGF | NM_001963.3 | | 2,7 | | 0,01 | 9,1 | 0,01 | 3,8 | | 0,02 |
| PLA2G4C | NM_003706.2 | | 2,4 | | 0,17 | 14,3 | 0,00 | 10,6 | | 0,02 |
| RELA | NM_021975.3 | | 1,2 | | 0,18 | 2,6 | 0,04 | -1,2 | | 0,42 |
| BMP8A | NM_181809.3 | | -1,5 | | 0,20 | 1,5 | 0,02 | -2,6 | | 0,15 |
| PPP2R1A | NM_014225.3 | | 1,1 | | 0,26 | -1,5 | 0,01 | -1,1 | | 0,44 |
| HDAC5 | NM_005474.4 | | -1,2 | | 0,28 | 1,5 | 0,03 | -1,5 | | 0,04 |
| COL4A5 | NM_033381.1 | | 1,3 | | 0,41 | 3,0 | 0,03 | -2,6 | | 0,03 |
| SPRY1 | NM_005841.1 | | 2,0 | | 0,41 | 8,5 | 0,01 | 2,9 | | 0,06 |
| ATM | NM_138292.3 | | 1,2 | | 0,44 | 2,6 | 0,02 | -1,1 | | 0,75 |
| BAMBI | NM_012342.2 | | -1,1 | | 0,49 | 1,8 | 0,02 | 1,1 | | 0,55 |
| IGFBP3 | NM_000598.4 | | 1,9 | | 0,51 | 6,9 | 0,03 | 1,1 | | 0,89 |
| PLA2G10 | NM_003561.1 | | -1,3 | | 0,53 | 2,3 | 0,03 | -2,2 | | 0,15 |
| LTBP1 | NM_000627.3 | | 1,2 | | 0,54 | 2,6 | 0,01 | -1,4 | | 0,18 |
| COL4A3 | NM_000091.3 | | -1,7 | | 0,56 | 2,0 | 0,00 | 1,2 | | 0,46 |
| PLA2G4E | NM_001206670.1 | | 1,3 | | 0,72 | -1,9 | 0,03 | 1,6 | | 0,44 |
| SPP1 | NM_000582.2 | | 1,3 | | 0,76 | 10,2 | 0,00 | 2,7 | | 0,02 |
| IL2RB | NM_000878.2 | | -1,1 | | 0,79 | -1,9 | 0,03 | 2,5 | | 0,10 |
| NGF | NM_002506.2 | | -1,1 | | 0,79 | 3,4 | 0,05 | -1,1 | | 0,84 |
| PAK7 | NM_177990.1 | | 1,1 | | 0,84 | -1,7 | 0,05 | 1,4 | | 0,66 |
| IL6 | NM_000600.1 | | 1,1 | | 0,84 | -1,9 | 0,03 | -1,1 | | 0,73 |
| DNMT1 | NM_001379.2 | | 1,0 | | 0,85 | -1,8 | 0,01 | -1,0 | | 0,93 |
| BIRC3 | NM_182962.1 | | -1,0 | | 1,00 | 7,8 | 0,00 | 1,3 | | 0,44 |

**Table S2.** 30 unique significantly dysregulated genes under SARB treatment.

| Gene Name | Accession # | Combi | p-value | SARB | p-value |
| --- | --- | --- | --- | --- | --- |
| Downregulated |  |  |  |  |  |
| DKK2 | NM_014421.2 | -1,9 | 0,18 | -8,5 | 0,03 |
| GRIN2A | NM_000833.3 | -2,4 | 0,22 | -7,4 | 0,02 |
| GATA1 | NM_002049.2 | 1,1 | 0,87 | -7,2 | 0,02 |
| HNF1A | NM_000545.4 | -1,3 | 0,62 | -4,5 | 0,04 |
| THBS4 | NM_003248.3 | -1,0 | 0,96 | -4,1 | 0,02 |
| RNF43 | NM_017763.4 | -1,2 | 0,57 | -3,1 | 0,03 |
| CREB3L4 | NM_130898.2 | -1,4 | 0,19 | -2,2 | 0,05 |
| CREB5 | NM_182898.2 | 1,7 | 0,06 | -2,1 | 0,04 |
| MAPK1 | NM_138957.2 | -1,5 | 0,11 | -2,1 | 0,03 |
| LEP | NM_000230.2 | -1,5 | 0,48 | -2,1 | 0,05 |
| PRMT8 | NM_019854.3 | -1,1 | 0,79 | -2,1 | 0,05 |
| TNN | NM_022093.1 | -1,8 | 0,11 | -2,1 | 0,05 |
| IL13RA2 | NM_000640.2 | -1,4 | 0,57 | -2,0 | 0,05 |
| HELLS | NM_018063.3 | -1,4 | 0,15 | -1,9 | 0,05 |
| CCND2 | NM_001759.2 | -1,5 | 0,32 | -1,8 | 0,05 |
| FUBP1 | NM_003902.3 | -1,2 | 0,16 | -1,8 | 0,01 |
| MAPK9 | NM_139068.2 | -1,4 | 0,06 | -1,8 | 0,01 |
| MDC1 | NM_014641.2 | -1,2 | 0,31 | -1,7 | 0,05 |
| SUV39H2 | NM_024670.3 | -1,4 | 0,04 | -1,7 | 0,01 |
| CDK4 | NM_000075.2 | -2,2 | 0,06 | -1,6 | 0,04 |
| CDKN2C | NM_001262.2 | -1,5 | 0,21 | -1,6 | 0,00 |
| FOS | NM_005252.2 | 5,1 | 0,00 | -1,6 | 0,05 |
| TFDP1 | NM_007111.4 | -1,1 | 0,24 | -1,5 | 0,01 |
| BMP8A | NM_181809.3 | 1,5 | 0,02 | -1,5 | 0,02 |
| BRAF | NM_004333.3 | -1,0 | 0,86 | -1,5 | 0,03 |
| Upregulated |  |  |  |  |  |
| PTCH1 | NM_000264.3 | 1,4 | 0,11 | 1,7 | 0,04 |
| WNT10B | NM_003394.2 | 1,1 | 0,74 | 1,6 | 0,05 |
| PPP2CB | NM_001009552.1 | 1,4 | 0,12 | 1,6 | 0,03 |
| SIX1 | NM_005982.3 | -1,3 | 0,71 | 2,7 | 0,05 |
| CC2D1B | NM_032449.2 | 4,9 | 0,25 | 5,8 | 0,03 |

**Table S3.** List of the 16 deregulated genes under Combi and SARB treatment included in the PI3K/AKT signalling pathway.

| Gene Name | Accession # | Combi | p-value | SARB | p-value |
| --- | --- | --- | --- | --- | --- |
| GNG7 | NM_052847.1 | -1,3 | 0,27 | -10,7 | 0,04 |
| AKT3 | NM_181690.1 | -1,3 | 0,27 | -7,6 | 0,00 |
| THBS4 | NM_003248.3 | -1,0 | 0,96 | -4,1 | 0,02 |
| **KITLG** | **NM_003994.4** | **-3,3** | **0,00** | **-3,5** | **0,00** |
| **RELN** | **NM_005045.2** | **-3,0** | **0,04** | **-2,7** | **0,05** |
| IL3 | NM_000588.3 | -2,5 | 0,16 | -2,6 | 0,04 |
| **EIF4EBP1** | **NM_004095.3** | **-2,9** | **0,04** | **-2,5** | **0,00** |
| **DDIT4** | **NM_019058.2** | **-1,7** | **0,04** | **-2,5** | **0,01** |
| KRAS | NM_004985.3 | -1,6 | 0,08 | -2,5 | 0,01 |
| MAPK3 | NM_001040056.1 | -1,0 | 0,92 | -2,3 | 0,05 |
| CREB3L4 | NM_130898.2 | -1,4 | 0,19 | -2,2 | 0,05 |
| CREB5 | NM_182898.2 | 1,7 | 0,06 | -2,1 | 0,04 |
| MAPK1 | NM_138957.2 | -1,5 | 0,11 | -2,1 | 0,03 |
| TNN | NM_022093.1 | -1,8 | 0,11 | -2,1 | 0,05 |
| CCND2 | NM_001759.2 | -1,5 | 0,32 | -1,8 | 0,05 |
| CDK4 | NM_000075.2 | -2,2 | 0,06 | -1,6 | 0,04 |

**Table S4**. List of primers used for RT-qPCR experiments (Metabion).

| Primers | Forward 5’-3’ | Reverse 5’ – 3’ |
| --- | --- | --- |
| AXIN2 | CTGGCTCCAGAAGATCACAAAG | ATCTCCTCAAACACCGCTCCA |
| DKK2 | CTCTGGATGGTACTCGGCAC | GGTCTCCTTCATGCCCTTTTA |
| FGF9 | CCTGGGTCAGTCCGAAGC | CAGAATGCCAAATCGGCTGTG |
| ID2 | ATCCTGTCCTTGCAGGCTTC | ACCGCTTATTCAGCCACACA |
| c-Myc | TGAGGAGACACCGCCCAC | CAACATCGATTTCTTCCTCATCTTC |
| FOS | GGGGCAAGGTGGAACAGTTA | AGTTGGTCTGTCTCCGCTTG |
| B2M | GACTTGTCTTTCAGCAAGGA | ACAAAGTCACATGGTTCACA |

**Spheroid formation assay ImageJ macro**

*run("Set Scale...", "distance=2.7 known=10 pixel=1 unit=µm global");*

*run("8-bit");*

*//run("Brightness/Contrast...");*

*run("Enhance Contrast", "saturated=0.35");*

*//Uses Yen thresholding algorithm*

*setAutoThreshold("Yen");*

*setOption("BlackBackground", false);*

*run("Convert to Mask");*

*getHistogram(0,hist,256);*

*ratio = hist[255]/hist[0];*

*if (ratio>1) {*

*// closes the image, reopens it, subtracts the background and proceeds as normal*

*close();*

*open(inputFolder + filename);*

*run("8-bit");*

*run("Subtract Background...", "rolling=50 light");*

*setAutoThreshold("Yen");*

*setOption("BlackBackground", false);*

*run("Convert to Mask");*

*run("Remove Outliers...", "radius=50 threshold=0 which=Dark");*

*getHistogram(0,hist,256);*

*ratio = hist[255]/hist[0];};*

*run("Maximum...", "radius=10");*

*run("Fill Holes");*

*run("Minimum...", "radius=10");*

*//small spheroids require a more "gentle" function to clean up noise*

*run("Median...", "radius=10");*

*run("Maximum...", "radius=10");*

*run("Minimum...", "radius=10");*

*run("Fill Holes");*

*run("Watershed");*

*run("Analyze Particles...", "size=50000-Infinity circularity=0.20-1.00 show=[Overlay Outlines] display exclude include summarize");};*

*if (ratio >=0.001 && ratio<0.01) {*

*run("Maximum...", "radius=10");*

*run("Fill Holes");*

*run("Minimum...", "radius=10");*

*//slightly bigger spheroids and a more rigorous function to remove noise*

*run("Remove Outliers...", "radius=10 threshold=0 which=Dark");*

*run("Watershed");*

*run("Analyze Particles...", "size=50000-Infinity circularity=0.20-1.00 show=[Overlay Outlines] display exclude include summarize");};*

*if (ratio>=0.01 && ratio<0.2) {*

*run("Maximum...", "radius=10");*

*run("Fill Holes");*

*run("Minimum...", "radius=10");*

*run("Remove Outliers...", "radius=50 threshold=0 which=Dark");*

*run("Median...", "radius=10");*

*run("Watershed");*

*run("Analyze Particles...", "size=50000-Infinity circularity=0.20-1.00 show=[Overlay Outlines] display exclude include summarize");};*

*if (ratio>=0.2 && ratio<1) {*

*//Very big spheroids generally do not need to be expanded much to fill up the edges.*

*run("Maximum...", "radius=10");*

*run("Fill Holes");*

*run("Minimum...", "radius=10");*

*//Outliers and noise are removed rigorously*

*run("Remove Outliers...", "radius=50 threshold=0 which=Dark");*

*run("Minimum...", "radius=10");*

*run("Maximum...", "radius=8");*

*run("Watershed");*

*run("Analyze Particles...", "size=50000-Infinity circularity=0.20-1.00 show=[Overlay Outlines] display exclude include summarize");};*

*if (Overlay.size > 0) {*

*//Sends particles detected to the ROI manager*

*run("To ROI Manager");*

*close()*

**Figure 3E original blot.** The complete original blot of Figure 3E in which two treatments (TQ and 5-FU) were excluded.

**
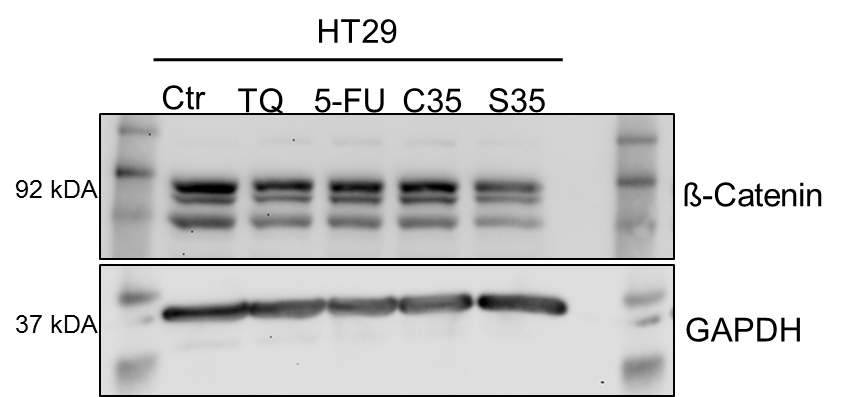
**

**References**

1 Effenberger, K., Breyer, S. & Schobert, R. Terpene conjugates of the Nigella sativa seed-oil constituent thymoquinone with enhanced efficacy in cancer cells. *Chem. Biodiv.* **7**, 129-139, doi:10.1002/cbdv.200900328 (2010).

2 Ouyang, L. *et al.* Selective bone targeting 5-fluorouracil prodrugs: synthesis and preliminary biological evaluation. *Bioorg. Med. Chem.* **19**, 3750-3756, doi:10.1016/j.bmc.2011.05.004 (2011).

3 Yusufi, M. *et al.* Synthesis, characterization and anti-tumor activity of novel thymoquinone analogs against pancreatic cancer. *Bioorg. Med. Chem. Lett.* **23**, 3101-3104, doi:10.1016/j.bmcl.2013.03.003 (2013).

4 Weiss, J. T. *et al.* Extracellular palladium-catalysed dealkylation of 5-fluoro-1-propargyl-uracil as a bioorthogonally activated prodrug approach. *Nature Commun.* **5**, 3277, doi:10.1038/ncomms4277 (2014).

5 Frohlich, T. *et al.* Synthesis of Novel Hybrids of Thymoquinone and Artemisinin with High Activity and Selectivity Against Colon Cancer. *ChemMedChem* **12**, 226-234, doi:10.1002/cmdc.201600594 (2017).

6 Kitamura, M., Koga, T., Yano, M. & Okauchi, T. Direct Synthesis of Organic Azides from Alcohols Using 2-Azido-1,3-dimethyl­imidazolinium Hexafluorophosphate. *Synlett* **23**, 1335-1338, doi:10.1055/s-0031-1290958 (2012).

7 Shao, C. *et al.* Acid-base jointly promoted copper(I)-catalyzed azide-alkyne cycloaddition. *The J. Org. Chem.* **76**, 6832-6836, doi:10.1021/jo200869a (2011).

**Figure Legends**

**Supplement Figure 1.** Combi and SARB treatment potentiate the cytotoxic effects of the single compounds. The cytotoxic effects of the individual compounds (5-FU and TQ), Combi and SARB on HCT116, HT29 and HCEC cells. **(A)** Cell viability of TQ, **(B)** 5-FU, **(C)** Combi and **(D)** SARB treatment in HCT116 and in HT29 cells. The data are presented as the mean percentage of the viability compared to DMSO controls. Error bars indicate SEM from the mean of three independent experiments each done in sixtuplicate. **E)** IC_50_ values of single compounds, Combi and the three hybrids in HCT116 and HT29 cells calculated at 48 h of treatment. IC_50_ values for AC29 and KV98 were calculated from two independent experiments. **(F)** HCEC cells were treated with increasing concentrations of SARB hybrid and cell viability was measured after 48 h of incubation. Error bars indicate SEM from the mean of two independent experiments each done in sixtuplicate (*p < 00.5, **p < 0.01; ***p < 0.001; One-way ANOVA).

**Supplement Figure 2.** All compound effects on organoids derived from mouse small intestine and the apoptotic evaluation via FACS. **(A)** Microscopic images of the morphology of wildtype mouse small intestinal organoids after treatment for 48 h with TQ, 5-FU, Combi and SARB at the indicated concentrations. The same organoids were monitored over time. Representative images are shown before treatment and after the incubation period. Scale bars-100 µm. **(B)** Cell viability was assessed using CellTiter-Glo 3D where the treated wells were compared to the DMSO control group. Data are calculated from two animals, each done in quintuplicate. From the ten values, the highest and the lowest were excluded. **(C)** FACS analysis of cell apoptosis in HCT116. Error bars indicate ± SEM from the mean of two independent experiments. **(D)** FACS analysis in HT29 cells after 48 h of treatment with TQ 40, 5-FU 15, Combi 35 and SARB 35 µM. Error bars indicate ± SEM from the mean of three independent experiments, (*p < 00.5, **p < 0.01; ***p < 0.001; One-way ANOVA). All graphs were generated using GraphPad Prism 7.0

**Supplement Figure 3.** Gene expression profiling under TQ and 5-FU individual treatments and the corresponding dysregulated pathways. **(A)** Scatter plot of normalized count values (log10) of dysregulated genes under TQ and 5-FU, following treatment for 24 h. All 770 genes were plotted. Pearson´s correlation was calculated using GraphPad Prism 7.0. **(B)** KEGG pathway analysis was performed using STRING database on the downregulated genes under TQ, **(C)** upregulated genes under TQ, **(D, E)** down and upregulated genes in 5-FU treatment, respectively. **(F, G)** The KEGG pathway analysis of upregulated genes under Combi and SARB, respectively. A p-value of ≤ 0.05 and a fold change of ≥ / ≤ 1.5-fold were selected for pathway analysis.

**Supplement Figure 4.** Combi and SARB treatment did not affect migration capability. **(A)** Wound closure calculated at 24 h and 48 h according to the formula given in material and methods (data shown as mean ± SEM of three independent experiments; n=3).

**Supplement Figure 5.** SARB hybrid inhibits the sphere propagation capacity of CRC HCT116 cells. **(A)** Schematic representation of the protocol used for propagation assay. **(B)** Representative bright-field images of generation one of the propagated cells. HCT116 cells treated with sub-doses of SARB are shown. **(C)** Quantification of propagated spheres after one generation was done using Image J. **(G)** Representative bright-field images of generation two, **(E)** three, **(F)** four and **(G)** five of the propagated cells. The sphere number is shown as mean ± SEM of three independent experiments (****p* < 0.001; One-way ANOVA). Scale bars-250 µm.

**Supplement Figure 6.** Combi treatment diminished the sphere formation capacity. **(A)** Representative bright-field images of spheroids in the untreated HCT116 cells and in Combi treated cells after 48 h of treatment. The images are representative of two independent experiments. Scale bar-250 µm.
